# Immunometabolic analysis of primary murine group 2 innate lymphoid cells: a robust step-by-step approach

**DOI:** 10.3389/fimmu.2025.1545790

**Published:** 2025-03-13

**Authors:** Sai Sakktee Krisna, Rebecca C. Deagle, Nailya Ismailova, Ademola Esomojumi, Audrey Roy-Dorval, Frederik Roth, Gabriel Berberi, Sonia V. del Rincon, Jörg H. Fritz

**Affiliations:** ^1^ Department of Physiology, Faculty of Medicine and Health Sciences, McGill University, Montréal, QC, Canada; ^2^ Segal Cancer Centre, Lady Davis Institute for Medical Research, Jewish General Hospital, Montréal, QC, Canada; ^3^ McGill University Research Centre on Complex Traits (MRCCT), Faculty of Medicine and Health Sciences, McGill University, Montréal, QC, Canada; ^4^ Dahdaleh Institute of Genomic Medicine (DIgM), McGill University, Montréal, QC, Canada; ^5^ Department of Microbiology and Immunology, Faculty of Medicine and Health Sciences, McGill University, Montréal, QC, Canada; ^6^ Division of Experimental Medicine, Faculty of Medicine and Health Sciences, McGill University, Montréal, QC, Canada; ^7^ Department of Oncology, Faculty of Medicine and Health Sciences, McGill University, Montréal, QC, Canada

**Keywords:** group 2 innate lymphoid cells (ILC2), immunometabolism, mitochondria, seahorse analysis, SCENITH (Single Cell ENergetic metabolism by profilIng Translation inhibition)

## Abstract

Group 2 Innate Lymphoid Cells (ILC2s) have recently been shown to exert key regulatory functions in both innate and adaptive immune response networks that drive the establishment and progression of type 2 immunity. Although mainly tissue resident, ILC2s and their crosstalk within tissue microenvironments influence metabolism at both the local and systemic levels. In turn, the energetic demand and metabolic status within these systems shape the diverse phenotypes and effector functions of ILC2s. Deciphering these metabolic networks in ILC2s is therefore essential in understanding their various roles in health as well as their associated pathophysiologies. Here we detail a framework of experimental approaches to study key immunometabolic states of primary murine ILC2s and link them to unique phenotypes and their corresponding functionality. Utilizing flow cytometry, Single Cell ENergetic metabolism by profilIng Translation inHibition (SCENITH), and the Seahorse platform we provide a framework that allows in-depth analysis of cellular bioenergetic states to determine the immunometabolic wiring of ILC2s. Connecting immunometabolic states and networks to ILC2 phenotypes and effector functions with this method will allow future in-depth studies to assess the potential of novel pharmaceutics in altering ILC2 functionality in clinical settings.

## 1 Introduction

Large extracellular helminths have been suggested to constitute a major evolutionary driving force of the effector mechanisms of type 2 immune responses, conferring protection from parasites invading barrier tissues (1, 2). However, the precise factors that shape the magnitude and quality of type 2 immune cell responses remain incompletely understood. Interestingly, chronic helminth infections have been shown to be associated with significant morbidity, including malnutrition, suggesting competition between parasites and host for metabolic resources, potentially leading to immunomodulatory consequences and alterations in protective type 2 immunity (3). An emerging body of evidence suggests that the immune system senses and utilizes nutrients and metabolites derived directly from the diet or produced by commensal or pathogenic microbes (4, 5). During the early phases of helminth infection, alarmin signals such as interleukin (IL)-25, IL-33, and thymic stromal lymphopoietin (TSLP) are released by non-hematopoietic cells in response to tissue damage and act to induce rapid proliferation and expansion of group 2 innate lymphoid cells (ILC2). Primarily found at mucosal barrier sites, ILC2 are transcriptionally and functionally poised innate type 2 effector immune cells that, once activated, can rapidly release large quantities of IL-5 and IL-13 to elicit eosinophilia, goblet cell hyperplasia, epithelial cell extrusion, and smooth muscle hypercontractility (6). Furthermore, it is gradually appreciated that ILC2 respond to changes in the richness and accessibility of microbially- as well as dietary-derived metabolites, such as vitamin A–derived retinoic acid (7), aryl-hydrocarbon receptor ligands (8), short chain fatty acids (9), succinate (10, 11), and iron (12). These findings suggest that ILC2, in addition to danger signals, are poised to respond to the broader metabolic milieu of barrier tissues (13). Moreover, nutrients are essential components, providing fundamental metabolic substrates for the production of energy to fuel protein translation, cellular proliferation and immune cell effector functions (4, 5). Indeed, the ability of ILC2 to induce rapid effector functions depend on the ability to engage cell-intrinsic metabolic pathways to catabolize glucose, fatty acids and amino acids. Like T cells (14), ILC2s undergo metabolic reprogramming to meet the high energy demand imposed by cell proliferation and the activation of effector functions (15, 16).

In the resting state, ILC2 utilize branched chain amino acids (BCAAs) and arginine to support mitochondrial oxidative phosphorylation (OXPHOS) to sustain homeostatic functions (17). ILC2 become highly proliferative following activation with IL-33, relying on glycolysis and mammalian target of rapamycin (mTOR) to produce type 2 cytokines (18), while continuing to fuel OXPHOS with amino acids to maintain cellular fitness and proliferation (17, 19). Metabolic reprogramming during ILC2 effector differentiation also requires increased anabolic metabolism, leading to increased lipid droplet (LD) formation and requiring fatty acid oxidation (FAO) to fuel pathogenic allergic airway inflammation (20–23). In addition, the formation of LDs is facilitated by glucose and was shown to be important to produce phospholipids that fuel ILC2 proliferation (21). Glucose was further shown to regulate the gene expression of diglyceride acyltransferase (DGAT1) and peroxisome proliferator-activated receptor gamma (PPARγ) through the mTOR pathway (21). While DGAT1 drives LD formation (21), PPARγ promotes lipid uptake through CD36 expression (24–26). The increase in free fatty acid (FFA) uptake induced by ILC2 activation (20, 23) is fueled by group V phospholipase A2 (PLA2G5)-expressing macrophages (27). In addition, PLA2G5 intrinsically impacts IL-33 release from macrophages and the expression of the FFA receptor GPR40 on ILC2 (27). Interestingly, Atg5 deficiency lowered fatty acid metabolism gene expression induced by IL-33 and impaired FAO, type 2 cytokine production and cell fitness (22), suggesting that autophagy-mediated catabolic pathways are critical to sustain ILC2 metabolism.

In addition to sustaining homeostatic functions (17), amino acid metabolism is essential for lung ILC2 activation. Arginine metabolism by arginase 1 (Arg1) is required to fuel aerobic glycolysis for ILC2 proliferation and cytokine production (28). ILC2 constitutively express high levels of multiple solute carriers, including Slc3a2, Slc7a5 and Slc7a8 (19), known to encode for large neutral amino acid transporter chains (29). Slc3a2 encodes the protein CD98 that heterodimerizes with other solute carriers to form active amino acid transporters, such as Slc7a5 and Slc7a8, that together form the surface amino acid transporters LAT1 and LAT2 (29). ILC2-intrinsic deletion of Slc7a5 and Slc7a8 impaired the proliferative and cytokine-producing capacity through tuning of mTOR signaling (19). In addition, catabolism of tryptophane (Trp) by the enzyme tryptophan hydroxylase 1 (Tph1) drives ILC2 effector functions (30). Moreover, methionine metabolism facilitates ILC2-driven inflammation through STAT3-dependent production of type 2 cytokines (31), underlining the various ways amino acid metabolism contributes to the regulation of ILC2 homeostasis, proliferation and cytokine production. Collectively, these insights highlight that fine-tuning metabolic pathways is critical for maintaining ILC2 homeostasis and regulating their effector functions, including cell fitness, proliferation and cytokine production. Here we detail a framework of experimental approaches to study key immunometabolic states of primary murine ILC2 and link them to phenotypes and functionality.

## 2 Materials and methods

### 2.1 Mice

C57BL/6J mice were originally purchased from The Jackson Laboratory (Bar Harbor, Maine, USA) and bred in-house at McGill University under specific pathogen-free conditions with ad libitum access to food and water. Experiments were conducted with adult female mice (aged 8-12 weeks) in accordance with the guidelines and policies of McGill University and the Canadian Council on Animal Care.

### 2.2 Isolation of bone marrow-derived group 2 innate lymphoid cells

**Figure 1:**
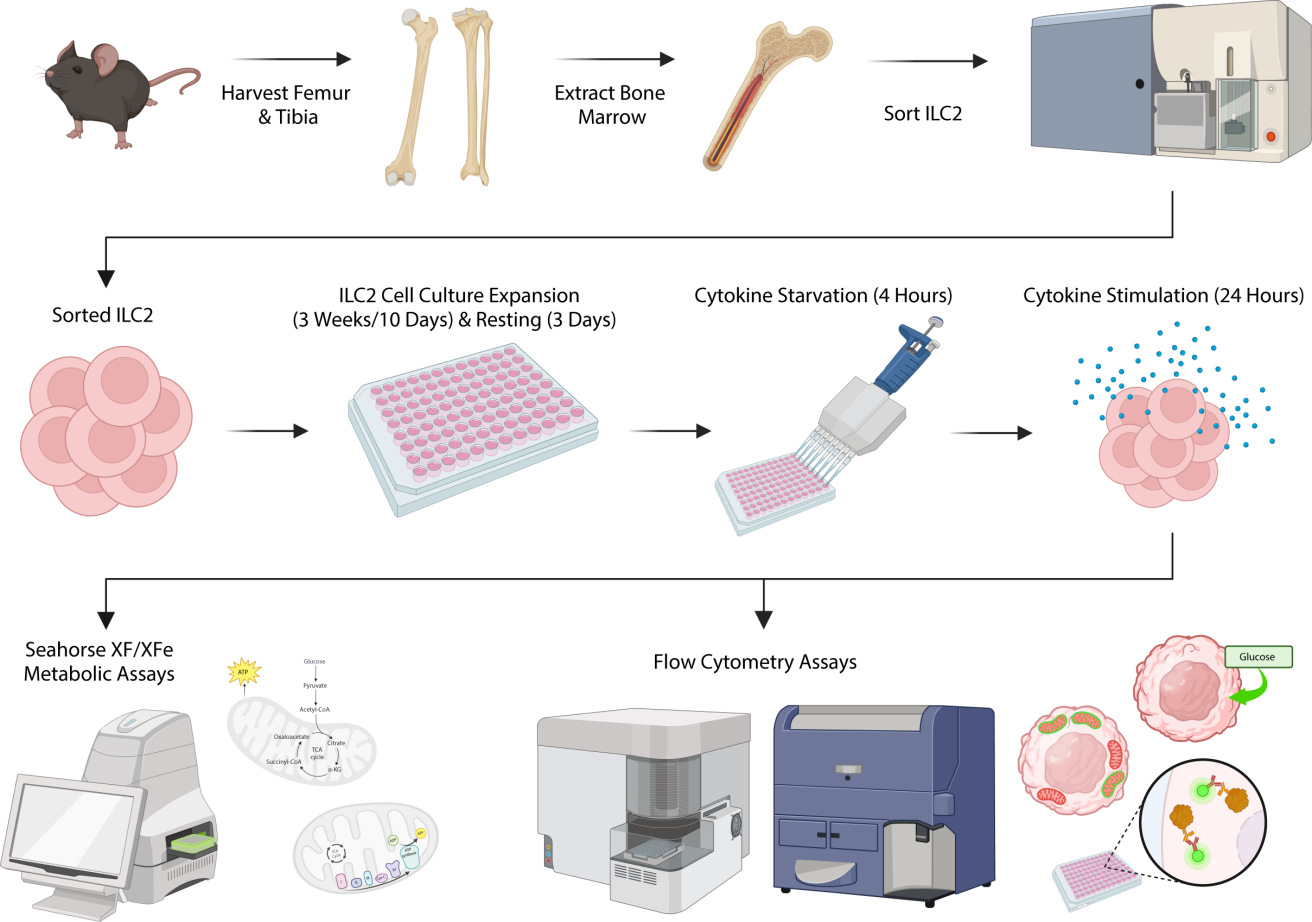
Schematic of experimental workflow. Femurs and tibias were harvested from female C57BL/6J wild-type mice and group 2 innate lymphoid cells (ILC2) were isolated from the bone marrow by flow cytometric cell sorting. ILC2 were cultured *in vitro* for expansion followed by a period of rest and cytokine starvation. ILC2 were subsequently re-stimulated with cytokines to determine metabolic activities using both flow cytometric assays and the Seahorse XF/XFe Analyzer. Created in BioRender. Deagle, R. (2025) https://BioRender.com/x72g976.

Adult female mice were anaesthetized with 5% isoflurane (Fresenius Kabi, Catalog No. CP0406V2) and euthanized via CO_2_ asphyxiation, followed by cervical dislocation as a confirmation of death. The hind legs were harvested, and the bulk of the muscles were removed with scissors to expose the leg bones. The femur and tibia bones were separated and cleaned with sterilized gauze (Fisher Scientific, Catalog No. 22-037-907) to remove any trace muscle or connective tissues, and briefly washed with 70% ethanol. Centrifugation was implemented to extract bone marrow from the femur and tibia bones. This centrifugation method began with the preparation of bone marrow extraction tubes, whereby a hole was punctured in the bottom of a 0.5 mL tube (Sarstedt, Catalog No. 72.737.002) with an 18-gauge needle (Becton Dickinson, Catalog No. 305196). These extraction tubes were sterilized by autoclave and each tube was set inside a sterile 1.5 mL collection tube (Progene, Catalog No. 87-B150-C) containing 100 µL of sterile Dulbecco’s Phosphate Buffered Saline (DPBS; Cytiva, Catalog No. SH30028.02). The bones were cut at the epiphysis on both ends to expose the bone marrow, and a single bone was vertically placed inside one 0.5 mL extraction tube. The 1.5 mL tubes containing the cut bones and extraction tubes were sealed and centrifuged at 1900*g for 5 minutes at room temperature. This allowed the bone marrow to be extracted through the punctured hole and into the collection tube with DPBS. After centrifugation, the 0.5 mL tubes and hollow bones were discarded. The bone marrow was resuspended in the DPBS and transferred to a 50 mL tube (Fisher Scientific, Catalog No. 14-432-22), with a maximum of 20 bones (5 mice) per tube. Each 50 mL tube was filled to a volume of 50 mL with DPBS and centrifuged at 450*g for 5 minutes at 4°C to wash the bone marrow. The supernatant was discarded, and the bone marrow pellet was resuspended in 1 mL of Ammonium-Chloride-Potassium (ACK) lysis buffer (ThermoFisher Scientific, Catalog No. A1049201) for 20 to 30 seconds to remove red blood cells from the pellet. This reaction was neutralized by filling the tube to 50 mL with Fluorescence-Activated Cell Sorting (FACS) buffer made with 2% Fetal Bovine Serum (FBS; Wisent, Catalog No. 080150) in DPBS. The tube was centrifuged, and the pellet was washed twice with FACS buffer (450*g, 5 minutes, 4°C) to remove any trace of the lysis buffer. To prevent non-specific binding of antibodies to the fragment crystallization receptors (FcRs), the cells were blocked for 15 minutes on ice using a 1:10 dilution of in-house formulated “Fc-block” (supernatant of the 2.4G2 hybridoma producing the purified anti-mouse CD16/CD32 monoclonal antibody (mAb)) in FACS buffer. The bone marrow cells were stained with an antibody cocktail (**Table 1**) for 30 minutes on ice in the dark directly in the blocking solution. The stained bone marrow cells were washed twice in FACS buffer (450*g, 5 minutes, 4°C), filtered through a 70 µm pore strainer (Fisher Scientific, Catalog No. 22-363-548), and kept on ice prior to cell sorting. Bone marrow-derived ILC2 were sorted using a FACS Aria III and FACSAria Fusion (BD Biosciences) equipped with 405 nm, 488 nm, 561 nm (Fusion only) and 640 nm lasers using FACSDiva Version 6.0 (Aria III) or Version 8.0 (Fusion) based on the absence of lineage markers and the expression of ILC2 specific markers (**Figure 1**, **Table 1**).

**Table 1:** Group 2 Innate Lymphoid Cell (ILC2) sorting antibodies.

Marker	Company	Fluorophore	Clone	Dilution	RRID or Catalogue Number
*Lineage Cocktail*					
TCRα/β	eBioscience	PE	H57-597	1:200	AB_466067
TCRγ/δ	eBioscience	PE	eBioGL3	1:400	AB_465934
CD3e	eBioscience	PE	145-2C11	1:400	AB_465497
Ly-6G/Ly-6C(Gr1)	eBioscience	PE	RB6-8C5	1:500	AB_466046
CD11b	eBioscience	PE	M1/70	1:500	AB_2734870
TER-119	eBioscience	PE	TER-119	1:400	AB_466043
CD45R (B220)	eBioscience	PE	RA3-6B2	1:200	AB_465672
CD19	eBioscience	PE	eBio1D3	1:200	AB_657660
NK1.1	eBioscience	PE	PK136	1:50	AB_466051
CD5	eBioscience	PE	53-7.3	1:200	AB_465524
CD11c	eBioscience	PE	N418	1:100	AB_465553
FceR1	eBioscience	PE	MAR-1	1:100	AB_466029
*ILC2^+^ Markers & Viability*					
Ly-6A/E (Sca-1)	BioLegend	Alexa Fluor 488	E13-161.7	1:200	AB_756201
CD25	eBioscience	eFluor 450	PC61.5	1:50	AB_10671550
CD117 (c-Kit)	eBioscience	APC	2B8	1:25	AB_469431
Viability	eBioscience	7-AAD	NA	1:60	00-6993-50

### 2.3 Expansion and resting of murine bone marrow-derived group 2 innate lymphoid cells

After cell sorting, isolated ILC2s were cultured in 96-well round-bottom plates (VWR, Catalog No. CA62406-121) at 37°C in 5% CO_2_ with a seeding density of 2.5x10^4^ cells per well in 200 µL of complete cell culture media (**Table 2**) with cytokines for expansion. These cytokines included: recombinant murine IL-2 (R&D Systems, Catalog No. 402-ML-100/CF), IL-7 (R&D Systems, Catalog No. 407-ML-200/CF), and IL-33 (R&D Systems, Catalog No. 3626-ML-010/CF) each at a concentration of 50 ng/mL and TSLP (R&D Systems, Catalog No. 555-TS-010/CF) at a concentration of 20 ng/mL. Every 2 days the cell culture was “split” whereby the cells were resuspended in the 200 µL volume, and 100 µL of the cell suspension was transferred to a new well. Each well was returned to a final volume of 200 µL by adding 100 µL of freshly prepared expansion cell culture media to facilitate proliferation. ILC2s were expanded for a maximum of three weeks, after which the cells were consolidated into 50 mL tubes and washed twice in RPMI 1640 (450*g, 5 minutes, 4°C) to remove any trace of expansion cytokines. The cells were counted and then seeded at a density of 2.5x10^4^ cells per well in 96-well round-bottom plates in 200 µL of complete cell culture media without β-mercaptoethanol (**Table 2**) and with 10 ng/mL each of IL-2 and IL-7. The cells incubated with these cytokines for three days at 37°C in 5% CO_2_ to “rest” the cells. This 3-day period of reduced activity was necessary after 3 weeks of energy-demanding proliferation to bring the cells down to a homeostatic baseline level of metabolic activity.

**Table 2:** Group 2 Innate Lymphoid Cell (ILC2) cell culture and assay media recipes.

Reagent	Company	Catalog Number	Final Concentration	Volume
*Complete Cell Culture Media*				
RPMI 1640 Medium	Cytiva	With Phenol Red SH30096.02 Phenol Red-Free SH30605.01		45 mL
Fetal Bovine Serum (FBS)	Wisent	080150	10%	5 mL
L-Glutamine (200 mM)	Cytiva	SH30034.01	2 mM	500 μL
Penicillin-Streptomycin (10,000 μg/mL)	Cytiva	SV30010	100 μg/mL	500 μL
Gentamicin (10 mg/mL)	Sigma-Aldrich	G1272	50 μg/mL	120 μL
β-Mercaptoethanol (55 mM) *Omitted for resting and experiments*	Gibco	21985023	50 μM	50 μL
*Agilent^TM^ Seahorse XF Cell Mito Stress and XF Real-Time ATP Rate Assay*				
Seahorse XF DMEM Medium	Agilent	103575-100		48.5 mL
Seahorse XF Glucose (1M)	Agilent	103577-100	10 mM	500 μL
Seahorse XF Pyruvate (100 mM)	Agilent	103578-100	1 mM	500 μL
Seahorse XF Glutamine (200 mM)	Agilent	103579-100	2 mM	500 μL
*Agilent^TM^ Seahorse XF Glycolysis Stress Assay*				
Seahorse XF DMEM Medium	Agilent	103575-100		49.5 mL
Seahorse XF Glucose (1 M)	Agilent	103577-100	2 mM	500 μL

### 2.4 Cell seeding and cytokine stimulation for experimental assays

After resting, ILC2s were consolidated into 50 mL tubes and washed twice in RPMI 1640 (450*g, 5 minutes, 4°C) to remove any trace of resting cytokines. Cells were counted and seeded at a density of 1.0x10^5^ in 100 μL of phenol red-free complete cell culture media (**Table 2**) either in a 96-well round-bottom plate (Sections 2.5-7, 2.10) or a Seahorse XFe96 cell culture microplate (Agilent™ Technologies, Catalog No. 103794-100; Section 2.8). Each condition for flow cytometric assays (Sections 2.5-7, 2.10) were plated in triplicate and each condition for Seahorse assays (Section 2.8) were plated in quintuplicate. The ILC2s incubated for 4 hours at 37°C in 5% CO_2_ without cytokines to starve the cells of cytokines, bringing them to a latent stage of activity prior to cytokine stimulation (**Figure 1**). The cytokine stimulation conditions for all experiments were the following: IL-2, IL-7, IL-33, IL-2+IL-33, and IL-7+IL-33. Each cytokine stimulation was prepared as a 2X concentrated solution (20 ng/mL) in phenol red-free complete cell culture media (**Table 2**). After cytokine starvation, 100 μL of the appropriate cytokine stimulation was added to the corresponding well for a total volume of 200 μL and a 1X concentration of cytokines (10 ng/mL). The ILC2s underwent cytokine stimulation for a period of 24 hours at 37°C in 5% CO_2_ prior to all experiments. After 24 hours of cytokine stimulation, ILC2 viability was typically lowest (78-80%) when stimulated with survival cytokines alone, such as IL-7 or IL-2, whereas ILC2 viability was highest (90-96%) when stimulated with activating cytokine conditions, such as IL-7+IL-33 or IL-2+IL-33.

### 2.5 Glucose uptake by flow cytometry

The glucose uptake in ILC2 was measured using the glucose analog 2-(N-(7-Nitrobenz-2-oxa-1,3-diazol-4-yl)Amino)-2-Deoxyglucose (2-NBDG; ThermoFisher Scientific, Catalog No. N13195) which was stored at -20°C at a stock concentration of 10 mM (5 mg lyophilised powder in 1.46 mL dimethyl sulfoxide (DMSO)). Immediately prior to experiments, the 10 mM stock of 2-NBDG was diluted 1:100 in warm phenol red-free complete media (**Table 2**) to a 2X concentrated solution of 100 μM. After 24 hours of cytokine stimulation, the cells were resuspended and transferred to a conical-bottom 96-well plate (Sarstedt, Catalog No. 82.1583.001). The cells were washed twice with phenol red-free RPMI 1640 (450*g, 5 minutes, 4°C) and resuspended in 100 μL of phenol red-free complete cell culture media (**Table 2**). A volume of 100 μL of the 2X concentrated 2-NBDG was added to each well (excluding unstained and viability controls) for a final 1X concentration of 50 μM, and the plate incubated with the glucose analog for 30 minutes at 37°C in 5% CO_2_. After incubation, the cells were washed once with phenol red-free RPMI 1640 and once with FACS buffer (450*g, 5 minutes, 4°C). The supernatant was discarded, and the cells were resuspended in 195 μL of FACS buffer prior to acquisition on the flow cytometer. Immediately before the sample was acquired on the flow cytometer, 5 μL of viability dye (7-AAD; eBioscience, Catalog No. 00-6993-50) was added to each sample (excluding the unstained control wells) and transferred to a 5 mL FACS tube (Fisher Scientific, Catalog No. 352008). Each sample was acquired using a LSRFortessa Cell Analyzer (BD Biosciences) until 20,000 events were recorded. 2-NBDG (465/540 nm) was acquired using the 488 nm laser and 7-AAD (535/617 nm) with the 561 nm laser. After sample acquisition, the files were exported in .fcs format from FACSDiva and analyzed using FlowJo software (BD, Version 10.10.0). Debris, doublet, and dead cells (7-AAD^+^) were excluded via flow cytometry gating. The 2-NBDG and the forward scatter were analyzed on the x- and y-axes, respectively. The reagent 2-NBDG had no adverse effect on the viability of ILC2 during this assay.

### 2.6 Analysis of PGC-1α expression levels by intracellular flow cytometry

**Viability staining, fixation, and permeabilization**. After 24 hours of cytokine stimulation, the cells were resuspended and transferred to a conical-bottom 96-well plate. The cells were washed twice with DPBS (450*g, 5 minutes, 4°C) and the supernatant was discarded. The fixable viability dye APC-eFluor780 (eBioscience, Catalog No. 65-0865) was stored at -80°C and diluted 1:1000 in DPBS. Excluding unstained controls, 50 μL of the diluted viability dye was added to each well and incubated on ice in the dark for 30 minutes. After incubation, the cells were washed twice with FACS buffer (450*g, 5 minutes, 4°C). The fixation/permeabilization solution was made during the centrifugation time, whereby the Fixation/Permeabilization Concentrate (eBioscience, Catalog No. 00-5123-43) was diluted 1:4 in Fixation/Permeabilization Dilutant (eBioscience, Catalog No. 00-5223-56). The supernatant from the cell culture plate was discarded, and 100 μL of the fixation/permeabilization solution was added to all wells and incubated on ice in the dark for 30 minutes. During incubation, the Permeabilization Buffer (10X; eBioscience, Catalog No. 00-8333-56) was diluted 1:10 in deionized water (dH_2_O). The fixed and permeabilized cells were washed twice with 100 μL of the 1X permeabilization buffer (600*g, 5 minutes, 4°C) and the 2.4G2 hybridoma blocking solution was prepared during the centrifugation time as previously described (Section 2.2). The supernatant from the cell culture plate was discarded and 50 μL of blocking solution was added to all wells, followed by 15-minute incubation on ice in the dark.

**Intracellular staining of PGC-1α.** While incubating cells in the blocking solution, the antibody dilutions were made in 1X permeabilization buffer. The PGC-1α antibody (Proteintech, Catalog No. CL488-66369) was diluted 1:1000 from its stock concentration of 1000 μg/mL to 1 μg/mL and the IgG1 isotype control antibody (Proteintech, Catalog No. CL488-66360-1) was diluted 1:200 from its stock concentration of 200 μg/mL to 1 μg/mL. After incubation, the cell culture plate was centrifuged (600*g, 5 minutes, 4°C) and the supernatant was discarded. A volume of 100 μL of antibody dilution was added to the appropriate wells, excluding unstained controls, and incubated for 30 minutes on ice in the dark. The stained cells were washed twice with 1X permeabilization buffer (600*g, 5 minutes, 4°C) and the supernatant was discarded. The cells were resuspended in 200 μL of FACS buffer and transferred to a 5 mL FACS tube.

**Flow cytometry.** Each sample was acquired using an Aurora Spectral Flow Cytometer (Cytek Biosciences) until 20,000 events were recorded. The directly conjugated PGC-1α and isotype control (493/522 nm) were acquired using the 488 nm laser and APC-eFluor780 (756/785nm) with the 633 nm laser. After sample acquisition, the files were exported in .fcs format from FACSDiva (BD Biosciences) and analyzed using FlowJo software (BD Biosciences, Version 10.10.0). Debris, doublet, and dead cells (APC-eFluor780^+^) were excluded via flow cytometry gating. The PGC-1α and the forward scatter were analyzed on the x- and y-axes, respectively.

### 2.7 Analysis of mitochondrial mass and membrane potential by flow cytometry

**Fluorescent label preparation.** Total mitochondrial mass was measured using Mitotracker™ Deep Red FM (ThermoFisher Scientific, Catalog No. M22426), which was stored at -20°C at a stock concentration of 1 mM (50 μg lyophilised powder in 91.98 μL DMSO). Mitochondrial membrane potential was measured using Tetramethylrhodamine, Methyl Ester, Perchlorate (TMRM; ThermoFisher Scientific, Catalog No. T668) which was stored at -20°C at a stock concentration of 10 mM (25 mg lyophilised powder in 5 mL DMSO). Ready-to-use TMRM aliquots were prepared at an intermediate concentration of 100 μM by diluting the stock 1:100 in DMSO and were stored at -20°C until needed for experiments. Immediately prior to experiments, the 1 mM Mitotracker™ Deep Red FM stock solution was diluted 1:10 in warm phenol red-free RPMI 1640 for an intermediate concentration of 100 μM. Both Mitotracker™ Deep Red FM and TMRM were finally diluted together 1:1000 in warm phenol red-free RPMI 1640 to a 2X concentrated solution of 100 nM for experimental conditions. Each dye was also prepared separately for single-variable controls. In addition, cell viability was measured using 4′,6-diamidino-2-phenylindole (DAPI; ThermoFisher Scientific, Catalog No. 62248) where only dead cell nuclei would be labeled. The 1 mg lyophilized stock was reconstituted in 1 mL of dH_2_O to make a 1 mg/mL stock solution and was stored at 4°C. An intermediate concentration of 100 μg/mL was made by diluting DAPI 1:10 in FACS buffer immediately prior to sample acquisition.

**Mitochondrial staining.** After 24 hours of cytokine stimulation, the cells were resuspended and transferred to a conical-bottom 96-well plate. The cells were washed twice with phenol red-free RPMI 1640 (450*g, 5 minutes, 4°C) and resuspended in 100 μL of phenol red-free complete cell culture media (**Table 2**). The 2X concentrated Mitotracker™ Deep Red FM and TMRM dilution was added to each well (excluding single color and viability controls) at a volume of 100 μL for a final volume of 200 μL and a final concentration of 50 nM for each dye. The cells incubated with the dyes for 30 minutes at 37°C in 5% CO_2_. This assay was optimized to use a concentration of 50 nM for each mitochondrial dye to ensure maximum viability, as overloading the cells with these dyes can create adverse effects. After incubation, the cells were washed once with phenol red-free media and once with FACS buffer (450*g, 5 minutes, 4°C). The supernatant was discarded, and the cells were resuspended in 198 μL of FACS buffer prior to acquisition on the flow cytometer.

**Flow cytometry and data export.** Immediately before the sample was acquired on the flow cytometer, 2 μL of the intermediate DAPI solution was added to each sample (excluding the unstained control wells) for a final concentration of 1 μg/mL and the cell suspension was transferred to a 5 mL FACS tube. Each sample was acquired using a LSRFortessa Cell Analyzer (BD Biosciences) until 20,000 events were recorded. Mitotracker™ Deep Red FM (644/665 nm) was acquired using the 633 nm laser, TMRM (548/573 nm) with the 561 nm, and DAPI (350/470 nm) with the 405 nm laser. After sample acquisition, the files were exported in .fcs format from FACSDiva and analyzed using FlowJo software. Debris, doublet, and dead cells (DAPI^+^) were excluded via flow cytometry gating. Mitotracker™ Deep Red FM and TMRM were analyzed on the x- and y-axes, respectively.

### 2.8 Agilent™ Seahorse Extracellular Flux Assay preparation

**Calibration and cell culture reagents.** Concurrent with the Seahorse XFe96 microplate preparation (Section 2.4), the base of the Seahorse XFe96 sensor cartridge (Agilent™ Technologies, Catalog No. 103792-100) was loaded with 200 μL of Seahorse XF Calibrant (Agilent™ Technologies, Catalog No. 100840-000) and incubated in a CO_2_-free incubator at 37°C for 24 hours to hydrate the lid of the sensor cartridge. The day of the experiment, assay media for the Seahorse experiments were freshly prepared and warmed to 37°C for experiments (**Table 2**). The Seahorse XF Mito Stress Assay and Seahorse XF Real-Time ATP Rate Assay used the same assay media composition where Seahorse XF DMEM medium was supplemented with L-glutamine, glucose, and pyruvate (**Table 2**). The Seahorse XF Glycolysis Stress Assay used the Seahorse XF DMEM medium supplemented with only L-glutamine (**Table 2**).

**Cell culture microplate.** After 24 hours of cytokine stimulation, the cell culture microplate was removed from the incubator and centrifuged at 450*g for one minute at room temperature. The 24-hour old cell culture media was carefully removed from the plate to minimize cell disruption and replaced with 180 μL of the freshly prepared assay media specific to the Seahorse assay being conducted (**Table 2**). The cell culture microplate incubated for one hour in a CO_2_-free incubator at 37°C. Incubation of both the sensor cartridge and the cell culture microplate in CO_2_-free conditions permitted proper diffusion of CO_2_ from the cells, medium, and plate; ensuring that all were properly de-gassed prior to running the assays.

**Stock solutions.** During this one-hour incubation time, the relevant assay media was used to make stock solutions of the different compounds associated with the Seahorse XF Mito Stress Test Kit (Agilent™ Technologies, Catalog No. 103015-100), the Seahorse XF Real-Time ATP Rate Assay Kit (Agilent™ Technologies, Catalog No. 103592-100), or the Seahorse XF Glycolysis Stress Test Kit (Agilent™ Technologies, Catalog No. 103020-100). The Seahorse XF Mito Stress Test Kit compounds included oligomycin, carbonyl cyanide-4 phenylhydrazone (FCCP), and rotenone + antimycin A; the Seahorse XF Real-Time ATP Rate Assay Kit included oligomycin and rotenone + antimycin A; and the Seahorse XF Glycolysis Stress Test Kit included glucose, oligomycin, and 2-Deoxy-Glucose (2-DG; **Table 3**). Each compound was resuspended several times by pipetting with the corresponding assay medium and were vortexed for one minute to ensure a thorough reconstitution of the compound (**Table 3**).

**Table 3:** The reconstitution volumes for each compound to generate the stock solutions from the Agilent^TM^ Seahorse XF Cell Mito Stress Assay, Real-Time ATP Rate Assay, and Glycolysis Stress Assay Kits.

Compound	Quantity per Tube (nmol)	Reconstitution Volume (with Assay Medium)	Stock Concentration	
*Agilent^TM^ Seahorse XF Cell Mito Stress Assay*				
Oligomycin	63	630 μL	100 μM	
FCCP	72	720 μL	100 μM	
Rotenone+Antimycin A	27 (each)	540 μL	50 μM	
*Agilent^TM^ Seahorse XF Real-Time ATP Rate Assay Kit*				
Oligomycin	63	420 μL	150 μM	
Rotenone+Antimycin A	27 (each)	540 μL	50 μM	
*Agilent^TM^ Seahorse XF Glycolysis Stress Assay Kit*				
Glucose	300,000	3000 μL	100 mM	
Oligomycin	72	720 μL	10 μM	
2-DG	1,500,000	3000 μL	500 mM	

Each compound reconstitution was completed with the assay medium specific to the assay.

**Loading concentrations.** The stock solutions were used to make a 10X concentrated solution of each compound with the corresponding assay media (**Table 4**). Each compound solution associated with the assay being performed was loaded into a specific port in the lid of the sensor cartridge (Port A, B, C, or D), which would be at a final 1X concentration after its timed injection during the assay (**Table 4**, Section 2.9). Any remaining compound solutions were discarded after the ports in the sensor cartridge lid were properly loaded. The Seahorse XFe96 sensor cartridge was returned to the CO_2_-free incubator until it was time to run the assay.

**Table 4:** The working concentration solutions and volumes of each compound for the Agilent^TM^ Seahorse XF Cell Mito Stress Assay, Real-Time ATP Rate Assay, and Glycolysis Stress Assay.

Reagent	Stock Solution Volume	Assay Media Volume	Loading Concentration (10X)	Volume Added to Port	Final Concentration (1X)
*Agilent^TM^ Seahorse XF Cell Mito Stress Assay*					
Oligomycin	450 μL	2550 μL	15 μM	20 μL (Port A)	1.5 μM
FCCP	600 μL	2400 μL	20 μM	22 μL (Port B)	2.0 μM
Rotenone/ Antimycin A	300 μL	2700 μL	5 μM	25 μL (Port C)	0.5 μM
*Agilent^TM^ Seahorse XF Real-Time ATP Rate Assay*					
Oligomycin	450 μL	2550 μL	15 μM	20 μL (Port A)	1.5 μM
Rotenone/ Antimycin A	300 μL	2700 μL	5 μM	22 μL (Port B)	0.5 μM
*Agilent^TM^ Seahorse XF Glycolysis Stress Assay*					
Glucose	3000 μL	0 μL	100 mM	20 μL (Port A)	10 mM
Oligomycin	300 μL	2700 μL	10 μM	22 μL (Port B)	1.0 μM
2-DG	3000 μL	0 μL	500 mM	25 μL (Port C)	50 mM

Each working concentration solution was made with the assay medium specific to the assay. The working concentrations were loaded into each port at a 10X concentration, achieving a 1X concentration after the timed injection during the assay runtime.

### 2.9 Agilent™ Seahorse Extracellular Flux Assay and data normalization

**Parameter setting and calibration.** The Seahorse XF/XFe Analyzer was turned on one hour prior to running the assays to stabilize. The Seahorse Wave Controller software (Agilent, Version 2.6) was opened on the computer, and the appropriate template file was selected (i.e.: Seahorse XF Cell Mito Stress Test, Glycolysis Stress Test, or Real-Time ATP Rate Assay). Under *Assay Navigation*, the option *Group Definitions* was selected and then *Groups* to create sample names. Sample names were determined based on the cytokine stimulations. A map of the conditions in the cell culture plate was generated under *Plate Map*, where wells labeled A1, A12, H1, and H12 were excluded to account for background signal. The sample names under *Group* were selected and applied to the generated plate map. A *Project Name* was created under *Run Assay* to identify the type and date of the assay, followed by *Start Run* to begin the calibration process. When the software prompted *Load Calibrant Utility Plate*, the sensor cartridge was removed from the CO_2_-free incubator and placed in the Seahorse XF/XFe Analyzer by selecting *Open Tray.* The option *I’m Ready* was selected to begin calibration which lasted approximately 20 minutes.

**Seahorse data acquisition.** When calibration was complete, the lid of the sensor cartridge was retained by the machine, and the base of the sensor cartridge was removed from the loading tray and replaced with the base of the cell culture microplate. The well labeled A1 in the cell culture microplate was aligned with the top-left corner of the loading tray and the option *Load Cell Plate* was selected to close the loading tray. The appropriate assay was chosen in the software to begin the assay. During the runtime of the assay, the Seahorse XF/XFe Analyzer automatically performed a timed injection of the relevant compound from the corresponding port in the lid of the sensor cartridge and into the cell culture plate below. The message *Unload Sensor Cartridge* signaled that the assay was complete, and the lid of the sensor cartridge was removed from the machine. *Eject* was selected to remove the cell culture microplate from the Seahorse XF/XFe Analyzer. The prompt *Assay Complete!* appeared on screen and the file was saved for viewing results after data normalization.

**Cell viability data acquisition.** The cell culture microplate was immediately used for cell viability quantification via flow cytometry to perform data normalization from the Seahorse assays. A volume of 5 μL of 7-AAD viability dye was added directly to each well, the cells were resuspended and transferred to a 5 mL FACS tube. Each sample was acquired using an Aurora Spectral Flow Cytometer (Cytek Biosciences) until 20,000 events were recorded. After sample acquisition, the files were exported in .fcs format from FACSDiva (BD Biosciences) and analyzed using FlowJo software (BD Biosciences, Version 10.10.0). Debris, doublet, and dead cells (7-AAD^+^) were excluded via flow cytometry gating and the number of live, single cells were exported as an Excel file (Microsoft Corporation, Version 16.88) in .xls format. Due to the nature of the assays interrupting metabolic processes and the length of time required to run the experiment, cell viability was lower than prior to the beginning of the assays.

**Seahorse data normalization and export.** The number of live, single cells were imported to a spreadsheet under *Normalize* in the Wave software. The Seahorse assay data was normalized according to the viability data by selecting *Apply.* To generate the data output from the assay, the *Results* tab was selected, followed by selecting the file of interest. In the *Functions* tab, *Export* was selected, and an Excel document was generated for each assay by selecting either Seahorse XF Cell Mito Stress Test Report Generator, Seahorse XF Glycolysis Stress Test Report Generator, or Seahorse XF Real-Time ATP Rate Assay Report Generator. Each Excel file was reviewed, and the three most consistent technical replicates (out of the five) were chosen from the dataset and transferred to GraphPad for graph configuration and statistical analyses (Section 2.13).

### 2.10 Agilent™ Seahorse Extracellular Flux Assay metric calculations

The following rationale and equations were used to quantify the rate of ATP production in ILC2s for both the oxidative phosphorylation (OXPHOS) and glycolytic metabolic pathways (Section 3.8). All equations, calculations, and contextual information were provided by the *Agilent™ Seahorse XF Real-Time ATP Rate Assay Kit User Guide*.

**Glycolytic ATP production rate calculation.** The Agilent™ Seahorse XF Real-Time ATP Rate Assay permits the measurement of the extracellular acidification rate (ECAR), determined by the extrusion of protons (H^+^) from the cells into the extracellular environment. The oxygen consumption rate (OCR) is measured simultaneously by the Seahorse XF/XFe Analyzer under basal conditions and continues to measure with each consecutive addition of mitochondrial inhibitors (oligomycin and rotenone/antimycin A). Pathway-specific information about mitochondrial or glycolytic ATP production (*mitoATP* and *glycoATP*, respectively) can be determined by transforming the ECAR and OCR data to ATP production rates (Section 3.8). In the glycolytic pathway, 2 molecules each of ATP, H^+^, and lactate are produced during the conversion of one glucose molecule:


(1) $$Glucose+2\;ADP+2\;Pi\;\to 2\;Lactate+2\;ATP+2\;H_{2}O+2\;H^{+}$$

Understanding the balance of Equation 1 and implementing the validated method previously described by Agilent™ (32) we can conclude, as shown in Equation 2, that the rate of ATP production in the glycolytic pathway (*glycoATP*) is equal to the Glycolytic Proton Efflux Rate (*glycoPER*):


(2) $$glycoATP\;Production\;Rate\;\left(\;pmol\;ATP/min\;\right)=glycoPER\;\left(\;pmol\;H^{+}/min\;\right)$$

**Mitochondrial ATP production rate calculation.** To calculate the ATP production rate associated with OXPHOS in the mitochondria (Equation 3), the difference is taken between the basal OCR and the OCR that is specifically inhibited by the ATP synthase inhibitor (oligomycin; *OCR_Oligo_*):


(3) $$OCR_{ATP}\;\left(\;pmolO_{2}/min\;\right)=OCR\;\left(\;pmolO_{2}/min\;\right)-OCR_{Oligo}\left(\;pmolO_{2}/min\;\right)$$

In turn, the *OCR_ATP_* needs to be transformed to the rate of mitochondrial ATP production (Equation 4). A multiplication of 2 is used to convert molecules of O_2_ to the number of oxygen (O) atoms consumed. This product is multiplied by the P/O ratio which represents “the number of molecules of ADP phosphorylated to ATP per atom of O reduced by an electron pair flowing through the electron transfer chain” (32). Agilent™ recommends an average value of 2.75, a standard P/O value for these calculations that was previously validated and accurately represents cell experimental conditions:


(4) $$\begin{array}{c} \;mitoATP\;Production\;Rate\;\left(\;pmol\;ATP/min\;\right)\\ =OCR_{ATP}\;\left(\;pmolO_{2}/min\;\right)\times 2\left(\;pmolO/min\;\right)\\ \times P/O\;(\;pmol\;ATP/pmolO\;) \end{array}$$

The total cellular ATP production (Equation 5) can finally be determined as the sum of both the glycolytic (Equation 2) and the mitochondrial (Equation 4) ATP production rates:


(5) $$\begin{array}{l} ATP\;Production\;Rate\;(pmol\;ATP/min)\\ \;=glycoATP\;Production\;Rate\;(pmol\;ATP/min)\\ \;+mitoATP\;Production\;Rate\;(pmol/min) \end{array}$$

### 2.11 SCENITH analysis of murine bone marrow-derived Group 2 Innate Lymphoid Cells

Single Cell ENergetic metabolism by profilIng Translation inHibition (SCENITH) analysis was performed with bone marrow-derived ILC2.

**Reagent preparation.** Glucose metabolism was inhibited with the glucose analog 2-Deoxy-D-Glucose (2-DG; Sigma Aldrich, Catalog No. D6134-25G) which was stored at -20°C at a stock concentration of 2 M (25 g crystalline powder in 76.14 mL dH_2_O). Mitochondrial ATP synthesis was inhibited with the antibiotic oligomycin (Sigma Aldrich, Catalog No. 75351-5MG) which was stored at -20°C at a stock concentration of 1 mM (5 mg lyophilized powder in 6.32 mL dH_2_O). The antibiotic puromycin (Sigma Aldrich, Catalog No. P7255-25MG) was used as a proxy for measuring protein synthesis, which was stored at -20°C at a stock concentration of 50 mg/mL (25 mg lyophilised powder in 500 µL dH_2_O). Immediately prior to experiments, each of these reagents were prepared as an intermediate 4X concentrated solution in complete cell culture media (**Table 2**). 2-DG was prepared at a concentration of 400 mM (1:5 dilution), oligomycin at 4 µM (1:250 dilution), and puromycin at 40 µg/mL (1:1250 dilution).

**Metabolic inhibition and sample preparation.** After 24 hours of cytokine stimulation, the cells were resuspended and transferred to a conical-bottom 96-well plate. The plate was centrifuged (450*g, 5 minutes, 4°C) and 100 µL of media was removed from each well, excepting the control wells which only removed 50 µL. In turn, 50 µL of the 4X concentrated solutions of either 2-DG, oligomycin, or a combination of the two were added to the respective wells for a total volume of 150 µL and incubated for 30 minutes at 37°C in 5% CO_2_. After incubation, 50 µL of the 4X puromycin was added to each well (excluding the negative control wells) for a final volume of 200 µL and incubated for an additional 15 minutes at the same conditions described above. The final 1X concentrations of 2-DG, oligomycin, and puromycin in the 200 µL volume were 100 mM, 1 µM, and 10 µg/mL, respectively.

**Antibody staining for puromycin (protein synthesis proxy) detection.** The cells were washed twice with DPBS (450*g, 5 minutes, 4°C) and then stained for viability with the fixable APC-eFluor780 dye, as described in Section 2.6. In brief, the cells incubated with the dye for 30 minutes on ice in the dark, followed by fixation, permeabilization, washes in 1X permeabilization buffer, and blocking in 2.4G2 hybridoma. The anti-puromycin antibody (Millipore-Sigma, Catalog No. MABE343-AF488) was diluted 1:1000 from its stock concentration of 0.5 mg/mL to 0.5 μg/mL. After incubation, the cell culture plate was centrifuged (600*g, 5 minutes, 4°C) and the supernatant was discarded. A volume of 100 μL of antibody dilution was added to the appropriate wells, excluding unstained controls, and incubated for 30 minutes on ice in the dark. The stained cells were washed twice with 1X permeabilization buffer (600*g, 5 minutes, 4°C) and the supernatant was discarded. The cells were resuspended in 200 μL of FACS buffer and transferred to a 5 mL FACS tube for flow cytometric acquisition.

**Flow cytometry.** Each sample was acquired using an Aurora Spectral Flow Cytometer (Cytek Biosciences) until 20,000 events were recorded. The directly conjugated anti-puromycin (493/522 nm) was acquired using the 488nm laser and APC-eFluor780 (756/785 nm) with the 633 nm laser. After sample acquisition, the files were exported in .fcs format from FACSDiva (BD Biosciences) and analyzed using FlowJo software (BD Biosciences, Version 10.10.0). Debris, doublet, and dead cells (APC-eFluor 780^+^) were excluded via flow cytometry gating. Anti-puromycin and forward-scatter were analyzed on the x- and y-axes, respectively. Cell viability was slightly lower than prior to the assay due to the interruption of metabolic processes, however the short exposure time (30 minutes) to metabolic inhibition did not severely compromise ILC2 integrity.

### 2.12 SCENITH metabolic calculations

The puromycin fluorescence (gMFI) in the cells measured by flow cytometry represents protein synthesis, and this output changes based on the presence of inhibitors that interrupt specific ATP producing processes. Due to the energy-demanding nature of protein synthesis, these data can be interpreted in terms of ATP production. Cells treated with 2-DG (DG) represent the ATP production when glycolysis is interrupted, oligomycin (O) represents ATP production when OXPHOS is interrupted, and cells treated with both inhibitors (DGO) represent ATP production when ATP synthesis is fully inhibited. These fluorescent signals are used in combination with control (Co) samples to calculate the specific dependencies and capacities of ILC2s.

**Glucose dependence.** The percentage of glucose dependence quantifies how dependent are translation levels on glucose oxidation. This is calculated as the difference in protein synthesis between control cells and those treated with 2-DG, divided by the difference between control cells and complete inhibition of ATP production (Equation 6):


(6) $$Glucose\;Dependence\;\left(\%\right)=100\times \left(\;\frac{Co-DG}{Co-DGO}\;\right)$$

**Mitochondrial dependence.** The percentage of mitochondrial dependence quantifies how much translation is dependent on OXPHOS, which is defined as the difference in protein synthesis between control cells and those treated with oligomycin, divided by the difference between control cells and complete inhibition of ATP production (Equation 7):


(7) $$Mitochondrial\;Dependence\;\left(\%\right)=100\times \left(\;\frac{Co-O}{Co-DGO}\;\right)$$

**Fatty acid oxidation (FAO) and amino acid oxidation (AAO) capacity.** The FAO & AAO capacity is defined as the capacity to use fatty acids and amino acids as sources for ATP production in the mitochondria when glucose oxidation is inhibited, including glycolysis and glucose-derived acetyl-CoA by OXPHOS (Equation 8):


(8) $$FAO\;\&\;AAO\;Capacity\;\left(\%\right)=100-\left(100\times \left(\frac{Co-DG}{Co-DGO}\right)\right)$$

**Glycolytic capacity.** The percentage of the glycolytic capacity is defined as the maximum capacity to sustain protein synthesis levels when mitochondrial OXPHOS is inhibited. Subtracting from 100 generates the value of how much ATP would be from glycolysis, without OXPHOS (Equation 9):


(9) $$Glycolytic\;Capacity\;\left(\%\right)=100-\left(100\times \left(\;\frac{Co-O}{Co-DGO}\;\right)\right)$$

### 2.13 Flow cytometric and statistical analysis

Flow cytometry analysis was performed using FlowJo software (BD, Version 10.10.0). Geometric mean fluorescence intensities (GeoMFI or gMFI) of the signals were calculated, triplicates were averaged per stimulatory condition and presented in bar graphs with all flow cytometry data represented as mean ± standard deviation (SD). Histograms and bar graphs were created using FlowJo and Prism softwares (Graphpad, Version 9) respectively. Statistical analysis was performed as ordinary one-way ANOVA and *post-hoc* Tukey’s multiple comparison tests to obtain statistical significance (*p*-values) between experimental conditions. *p*-values below 0.05 were defined as statistically significant (*p ≤ 0.05, **p ≤ 0.01, ***p ≤ 0.001, ****p ≤ 0.0001).

## 3 Results

The fine-tuning of metabolic pathways is critical for maintaining ILC2 homeostasis and the regulation of cellular fitness and effector functions. We therefore aimed to establish a comprehensive set of rapidly applicable experimental approaches to study metabolic activities of ILC2 using flow cytometry-based assays in combination with the Seahorse Analyser (**Figure 1**). The applied methods here detail the level of glucose uptake, the magnitude of energy production obtained by glycolysis and oxidative phosphorylation, as well as the plasticity of ILC2 metabolic programming of these respective pathways. Furthermore, we addressed numerous facets of mitochondrial involvement in ILC2 metabolism including mitochondrial biogenesis, the total mass of mitochondria per cell, the respiration of mitochondria, and the performance of that respiration.

### 3.1 Determining glucose uptake by ILC2

To study the metabolic activities of ILC2, we obtained primary murine sort purified bone marrow-derived ILC2 (**Figure 1**, **Supplementary Figure 1**) that were further expanded *in vitro* as previously described (33, 34). IL-33 has been established as a key driver of ILC2 activation (35). IL-7 and IL-2 secreted by tissue-resident non-hematopoietic stromal cells as well as innate and adaptive immune cells, respectively, have been shown to act synergistically with IL-33 to induce proliferation and cytokine production (33, 36, 37). However, the effects of these cytokines (IL-2, IL-7, IL-33) alone or in synergy (IL-2+IL-33, IL-7+IL-33) as they pertain to glucose metabolism in ILC2 remain incompletely understood.

**Figure 2:**
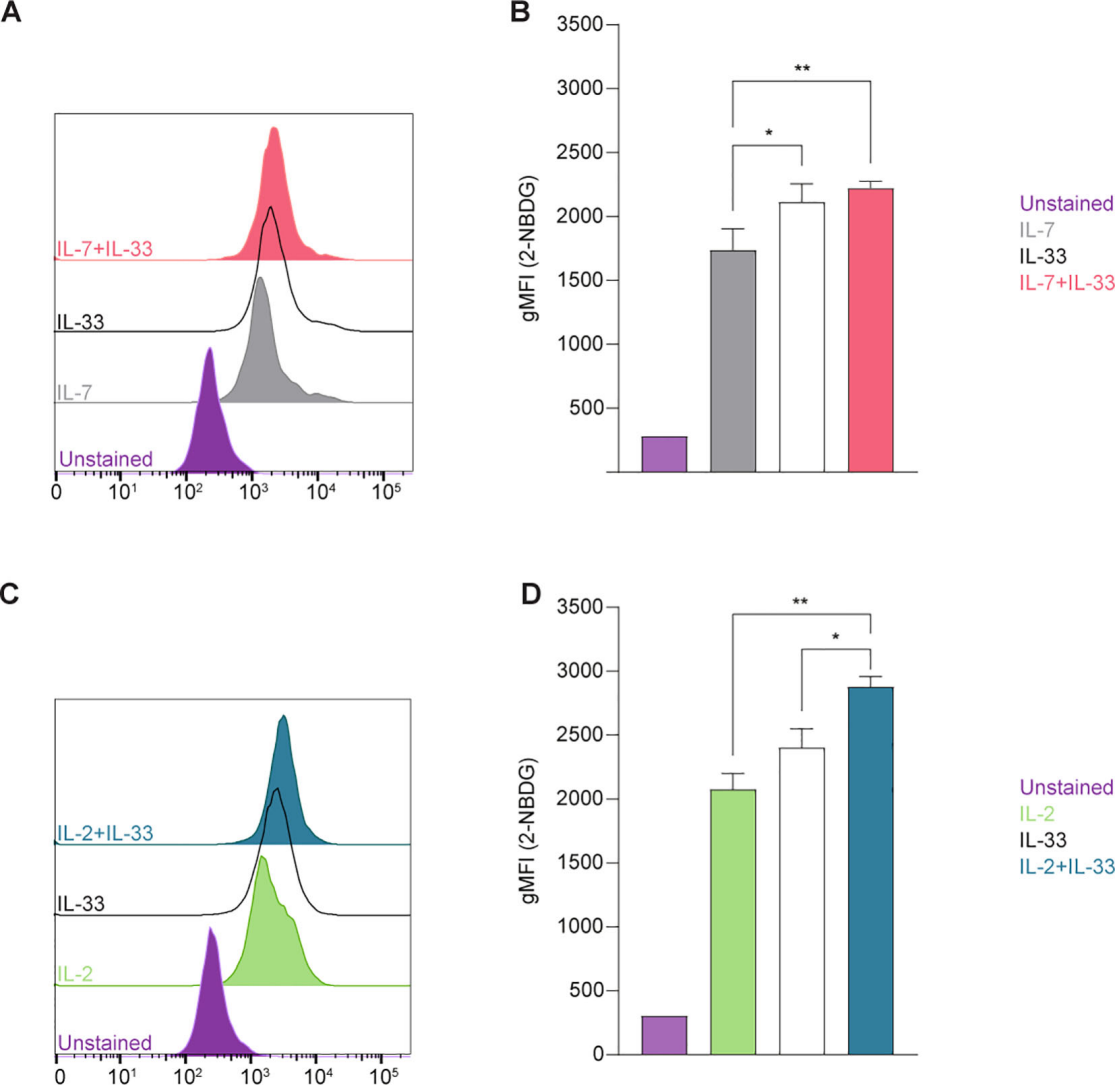
Group 2 innate lymphoid cells (ILC2) increase glucose uptake upon stimulation with activating cytokines. Bone marrow-derived group 2 innate lymphoid cells (ILC2) were stimulated with either IL-7 only, IL-33 only, or a combination of IL-7 and IL-33 **(A, B),** or IL-2 only, IL-33 only, or a combination of IL-2 and IL-33 **(C, D)**. All cytokines were applied at 10 ng/mL. 2-NBDG was added after 24 hours of cytokine stimulation and incubated for 30 minutes to assess capacity of glucose uptake by flow cytometric analysis, determining the geometric Mean Fluorescence Intensity (gMFI). ILC2 that were not incubated with 2-NBDG served as negative control (Unstained). Data reporting the treatment with IL-33 are the same for **(A-D)**. Data are shown as average ± standard deviation (SD) and are representative of three independent experiments. Statistical analysis was performed using one-way ANOVA followed by Tukey’s multiple comparisons test (p < 0.05 = *, p < 0.01 = **).

We first aimed to establish glucose uptake by ILC2 at defined steady and activation states using 2-(*N*-(7-Nitrobenz-2-oxa-1,3-diazol-4-yl)Amino)-2-Deoxyglucose (2-NBDG), a fluorescent glucose analogue (**Figure 2**). To investigate how activating cytokines impact glucose uptake, bone marrow-derived ILC2 were incubated with IL-7, IL-2, IL-33 alone, or in combinations of IL-7+IL-33 or IL-2+IL-33. After 24 hours of cytokine stimulation, 2-NBDG was added to the cells, incubated for 30 minutes, and subsequently analyzed by flow cytometry. Bone marrow-derived ILC2 treated with IL-7 (**Figures 2A, B**) or IL-2 alone (**Figures 2C, D**) exhibited moderate levels of exogenous glucose uptake, which considerably increased when treated in combination with IL-33. ILC2 treated with IL-33 alone displayed a higher uptake of exogenous glucose compared to IL-7 alone (**Figures 2A, B**), but not in regards to IL-2 alone (**Figures 2C, D**). In fact, the combination of IL-2+IL-33 exhibited the highest uptake of glucose compared to IL-33 or IL-2 alone (**Figures 2C, D**). Collectively, these observations demonstrate that ILC2 are actively acquiring exogenous glucose at steady state but were found to take up the most glucose when synergistically activated by IL-2+IL-33.

### 3.2 Quantification of mitochondrial biogenesis

**Figure 3:**
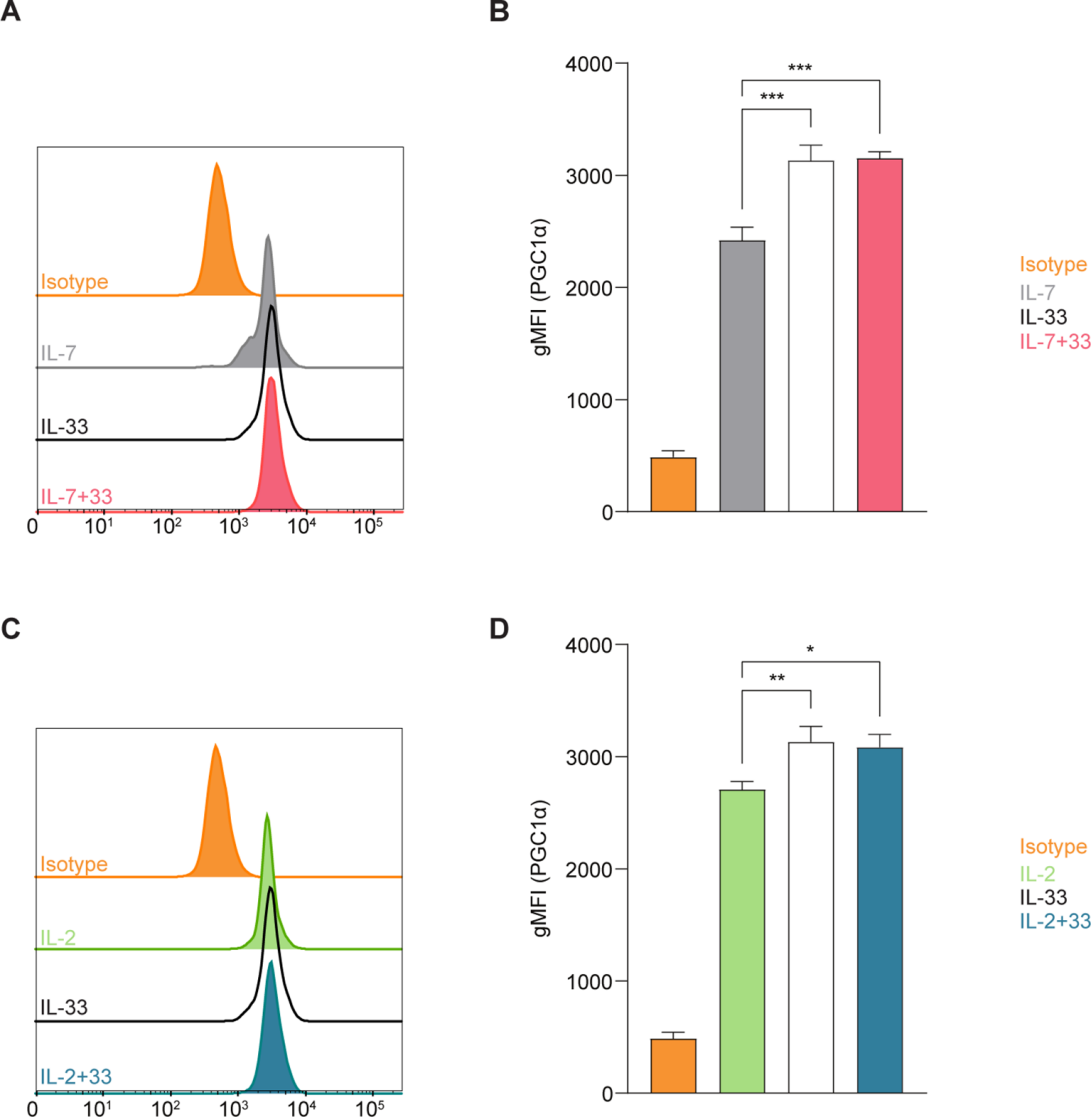
Group 2 innate lymphoid cells (ILC2) elevate expression of PGC-1a upon stimulation with activating cytokines. Bone marrow-derived group 2 innate lymphoid cells (ILC2) were stimulated with either IL-7 only, IL-33 only, or a combination of IL-7 and IL-33 **(A, B),** or IL-2 only, IL-33 only, or a combination of IL-2 and IL-33 **(C, D)**. All cytokines were applied at 10 ng/mL. After 24 hours of cytokine stimulation cells were harvested and the protein expression levels of the peroxisome proliferator–activated receptor gamma coactivator-1 alpha (PGC-1a) were assessed by intracellular flow cytometric analysis, determining the geometric Mean Fluorescence Intensity (gMFI). Isotype antibody stainings served as control (Isotype). Data reporting the treatment with IL-33 are the same for **(A-D)**. Data are shown as average ± standard deviation (SD) and are representative of three independent experiments. Statistical analysis was performed using one-way ANOVA followed by Tukey’s multiple comparisons test (p < 0.05 = *, p < 0.01 = **, p < 0.001 = ***).

Mitochondria are biosynthetic and bioenergetic organelles that also act as critical signaling platforms instructing decisions about cell proliferation, death, and differentiation. Mitochondria sustain immune cell phenotypes and functions, and depending on metabolic demands they can switch from being primarily catabolic ATP-generating organelles to anabolic organelles that produce the critical building blocks needed for macromolecule synthesis (38). Immune cells typically exhibit quiescent levels of metabolic activity at steady state and can shift to being highly metabolically active during the activation phase (5). This high energy demand triggers mitochondrial biogenesis to stimulate the production of more mitochondria and to replace mitochondria damaged by oxidative stress (39). The co-activator peroxisome proliferator-activated receptor gamma coactivator 1 alpha (PGC-1α) is characterized as a master regulator of mitochondrial biogenesis and oxidative metabolic pathways at both the transcriptional and post-translational levels (40). To investigate how cytokines known to drive ILC2 effector functions influence mitochondrial biogenesis, we first evaluated the expression levels of PGC-1α using intracellular flow cytometry. Bone marrow-derived ILC2 were incubated with IL-7, IL-2, IL-33 alone, or in combinations of IL-7+IL-33 or IL-2+IL-33. After 24 hours of cytokine stimulation, the cells were fixed, permeabilized, and labeled using a directly conjugated antibody for PGC-1α followed by flow cytometric analysis. ILC2 treated with IL-7 (**Figures 3A, B**) or IL-2 alone (**Figures 3C, D**) exhibited moderate levels of PGC-1α expression, which considerably increased in the presence of IL-33. There was no significant elevation in PGC-1α expression between IL-33 alone and the combination of either IL-7+IL-33 (**Figures 3A, B**) or IL-2+IL-33 (**Figures 3C, D**). However, these synergistic combinations of activating cytokines displayed higher levels of PGC-1α expression compared to IL-7 (**Figure 3B**) or IL-2 alone (**Figures 3C, D**). These data suggest that the presence of IL-33 primarily influences PGC-1α expression to drive mitochondrial biogenesis rather than relying on the synergistic activation of cytokines known to influence ILC2 effector functions.

### 3.3 Quantification of mitochondrial mass and active mitochondria

We next aimed to investigate mitochondrial metrics directly through the use of fluorescent functional dyes to be analyzed by flow cytometry. With the development of dyes such as MitoTracker™ Deep Red, it is possible to label all mitochondria within living cells and quantify the total mass produced by ILC2 at defined steady and activation states. MitoTracker™ Deep Red is a lipophilic carbocyanine-based dye that permeates the cell membrane to covalently bind thiol-reactive chloromethyl groups within the mitochondrial membrane, permitting all mitochondria to be fluorescently labeled (41). When glucose is acquired exogenously by the cell, the glucose is utilized by the mitochondria either through glycolysis or oxidative phosphorylation to produce energy in the form of adenosine triphosphate (ATP). While anaerobic glycolysis generates two ATP per glucose molecule, the aerobic process of oxidative phosphorylation produces 36 molecules of ATP (42). In brief, oxidative phosphorylation utilizes the electron transport chain (ETC) to drive protons (H^+^) against their concentration gradient out of the inner mitochondrial membrane space (42). This accumulation of H^+^ in the intermembrane space can then flow back through the ATP-generating component of the ETC, completing the energy production cycle (43). This difference in H^+^ concentration effectively creates both a pH and electrical gradient to generate a membrane potential in the mitochondria (42). This membrane potential, or polarization, can be exploited to label actively respirating mitochondria with other functional dyes, such as tetramethylrhodamine methyl (TMRM). TMRM is similar to MitoTracker™ Deep Red in that they are both lipophilic cationic dyes so they will both be drawn into mitochondria across this charged gradient, however, TMRM exhibits a low binding affinity to mitochondrial proteins and functional groups (43). Effectively, MitoTracker™ Deep Red labels all mitochondria that are present, whereas TMRM preferentially labels actively respirating mitochondria, and neither interacts with damaged mitochondrial membranes where this gradient is impaired.

**Figure 4:**
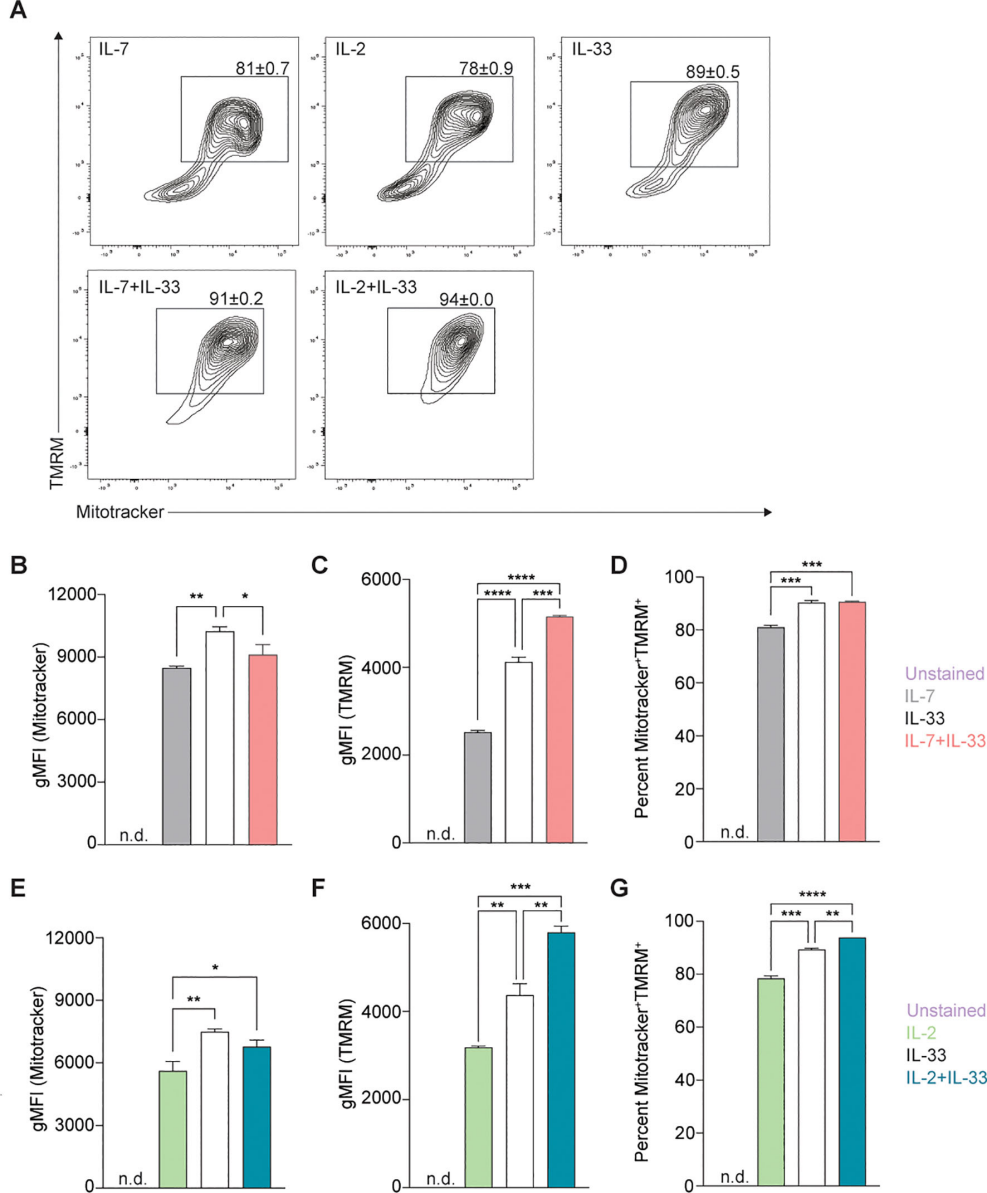
Stimulation of Group 2 innate lymphoid cells (ILC2) with activating cytokines augments mitochondrial mass and mitochondrial membrane polarization. Bone marrow-derived group 2 innate lymphoid cells (ILC2) were stimulated with either IL-7 only, IL-33 only, or a combination of IL-7 and IL-33 **(A, B-D)**, or IL-2 only, IL-33 only, or a combination of IL-2 and IL-33 **(A, E-G)**. All cytokines were applied at 10 ng/mL. After 24 hours of cytokine stimulation cells were harvested and stained with Mitotracker and TMRM to quantify by flow cytometric analysis mitochondrial mass as well as mitochondrial membrane potential, respectively. ILC2 not stained with MitoTracker and TMRM were used as negative control (Unstained). Flow cytometric contour plots are depicted in **(A)** with gates set on TMRM^+^ (y-axis) and MitoTracker^+^ (x-axis) double-positive populations. From gated populations geometric Mean Fluorescence Intensities (gMFI) for Mitotracker **(B, E)** and TMRM **(C, F)**, as well as frequencies of Mitotracker^+^TMRM^+^ double-positive populations **(D, G)** were determined. Data reporting the treatment with IL-33 are the same for **(A–G)**. Data are shown as average ± standard deviation (SD) and are representative of three independent experiments. Statistical analysis was performed using one-way ANOVA followed by Tukey’s multiple comparisons test (*p ≤ 0.05, **p ≤ 0.01, ***p ≤ 0.001, ****p ≤ 0.0001).

To further analyze whether mitochondrial biogenesis was replacing potentially damaged mitochondria or increasing the overall mass during activation states, we optimized a flow cytometry-based protocol to rapidly quantify the total mass of mitochondria in live bone marrow-derived ILC2. To investigate how activating cytokines impact the overall mass of mitochondria synthesized by ILC2, cells were incubated with IL-7, IL-2, IL-33 alone, or in combinations of IL-7+IL-33 or IL-2+IL-33. After 24 hours of cytokine stimulation, the mitochondrial dyes MitoTracker™ Deep Red and TMRM were added to the cells, incubated for 30 minutes, and ILC2s were subsequently analyzed by flow cytometry (**Figure 4A**). To investigate mitochondrial mass, we first analyzed ILC2 positive for MitoTracker™ Deep Red. ILC2 treated with IL-7 (**Figure 4B**) or IL-2 alone (**Figure 4E**) exhibited moderate levels of mitochondrial mass, which considerably increased in the presence of IL-33 alone. While there was no significant elevation in mitochondrial mass between IL-7 alone and the synergistic combination of IL-7+IL-33 (**Figure 4B**), there was a slight increase between IL-2 alone and IL-2+IL-33 (**Figure 4E**). Interestingly, the mitochondria mass decreased between IL-33 alone and IL-7+IL-33 (**Figure 4B**). These results suggest, similarly to the PGC-1α expression levels, that IL-33 influences the mitochondrial mass produced by ILC2 more than the synergistic effects of activating cytokines.

In addition, we sought to understand how active this mitochondrial mass was by investigating their membrane polarization as an indicator of mitochondrial respiration. To further understand how cytokines known to activate ILC2 impact mitochondrial respiration and activity, we analyzed bone marrow-derived ILC2 positive for TMRM to assess mitochondrial membrane polarization. Bone marrow-derived ILC2 treated with IL-7 (**Figure 4C**) or IL-2 alone (**Figure 4F**) exhibited low levels of membrane polarization, which considerably increased in the presence of IL-33. Furthermore, combined cytokine treatment of IL-7+IL-33 (**Figure 4C**) or IL-2+IL-33 (**Figure 4F**) markedly increased polarization of the mitochondrial membrane compared to either IL-7, IL-2, or IL-33 alone. These observations demonstrate that ILC2 exhibit low levels of mitochondrial polarization at steady state, actively increase their polarization upon treatment with IL-33, but especially when synergistically activated by IL-2+IL-33, or IL-7+IL-33.

Independent analysis of MitoTracker™ Deep Red and TMRM yielded information about the total mitochondrial mass and the total mitochondrial membrane polarization present within each cytokine stimulation, respectively. However, the information regarding what proportion of the total mitochondrial mass was actively respirating during defined steady and activation states remained unknown. Through the implementation of gating strategies (**Figure 4A**), we identified mitochondria that were positive for both MitoTracker™ Deep Red and TMRM to elucidate this ratio. Approximately 80% of the mitochondrial population was active in IL-7 (**Figure 4D**) or IL-2 alone (**Figure 4G**), which significantly increased to roughly 90% in the presence of IL-33. Similarly, compared to stimulations with IL-7 (**Figure 4D**) or IL-2 alone (**Figure 4G**), a marked elevation in the proportion of active mitochondria was found when ILC2 were activated by IL-7+IL-33 (**Figure 4D**) or IL-2+IL-33 (**Figure 4G**). However, while there was a significant increase in the proportion of active mitochondria between IL-33 alone and IL-2+IL-33 (**Figure 4G**), this was not the case when comparing stimulations of IL-33 with IL-7+IL-33 (**Figure 4D**). Collectively, these observations suggest that while mitochondrial mass production plateaus during peak ILC2 activation states, the mitochondrial membrane potential and overall activity increases when synergistically activated by IL-2+IL-33.

### 3.4 Determining cell density for Seahorse Extracellular Flux Assays

We next set out to complement flow cytometry-based assays with the Agilent™ Seahorse XF technology to assess specific inquiries about ILC2 glucose metabolism using the Mito Stress Assay, the Glycolysis Stress Test, and the Real-Time ATP assay. These approaches generate incredibly useful data that cover a myriad of metrics; however, they are also extremely sensitive assays that are heavily influenced by cell density. As such, the number of cells present in each well play a significant role in the detection of specific measurements, as well as the consistency between replicates. If the cellular confluence within the well is too dense, this will translate to measurements that are beyond the Seahorse XF/XFe Analyzer’s detection range resulting in inaccurate readings. Likewise, cellular confluence that is too sparse will result in measurements too low to be within the detection threshold. The primary measurement that is acquired in the Mito Stress Assay is called the Oxygen Consumption Rate (OCR), and implements the use of four compounds that modulate and probe mitochondrial functions: oligomycin, carbonyl cyanide-4 (trifluoromethoxy) phenylhydrazone (FCCP), rotenone, and antimycin A. In brief, the basal OCR is measured before the injection of oligomycin, the maximum OCR value is measured after the injection of FCCP, and the lowest OCR is measured after the injection of rotenone and antimycin A. According to Agilent’s user guide *Characterizing Your Cells: Using OCR Values to Determine Optimal Seeding Density*, a cellular confluence between 50-90% and a basal OCR range of 20-160 pmol/min is recommended for optimal results. However, these recommendations are general and not specific to ILC2. Here, we implemented the Mito Stress Assay to determine the optimal seeding density for OCR detection, even at the ILC2s highest and lowest OCR measurements across all cytokine stimulations.

**Figure 5:**
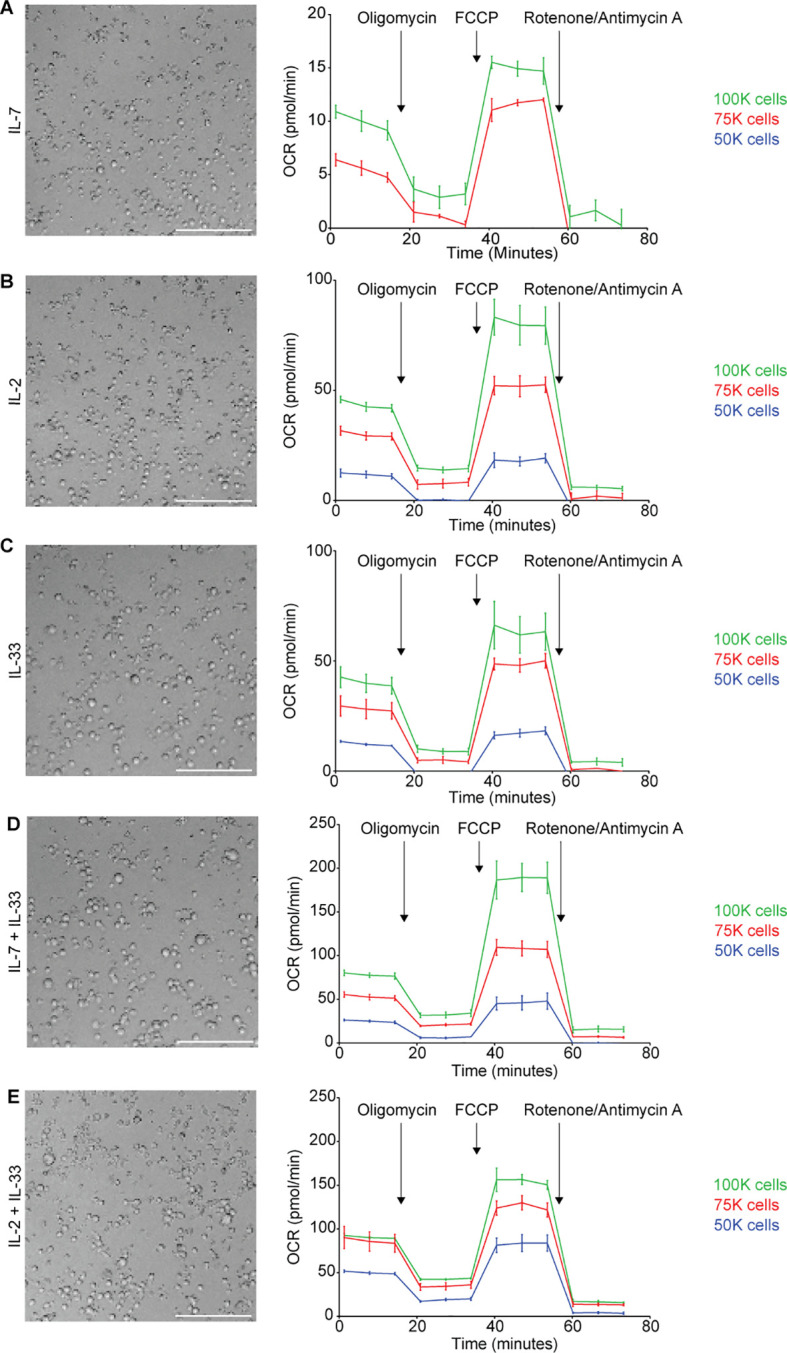
Determining cell seeding density for Seahorse metabolic assays. Bone marrow-derived group 2 innate lymphoid cells (ILC2) were seeded at three different cell densities (100.000 (100k), 75.000 (75k) or 50.000 (50k) per well) into Seahorse XFe96 microplates and stimulated with either IL-7 only **(A)**, IL-2 only **(B)**, IL-33 only **(C),** IL-7 and IL-33 **(D)**, or with IL-2 and IL-33 **(E)**. All cytokines were used at 10 ng/mL. After 24 hours of cytokine stimulation the Mito Stress Test assay was performed using the Seahorse Analyzer and oxygen consumption rates (OCR) were determined. Brightfield microscopy images of seeding densities of 100.000 cells/well are shown **(A-E)**; size bars = 150mM. Data are shown as average ± standard deviation (SD) and are representative of three independent experiments.

We performed a titration of three different cell concentrations that were seeded and incubated for 24 hours with either IL-7, IL-2, IL-33 alone or combinations of IL-7+IL-33, as well as IL-2+IL-33. The cell seeding densities of 50,000, 75,000 or 100,000 cells per well were used in the Mito Stress Assay to obtain OCR values (**Figure 5**). The seeding density of 50,000 cells/well resulted in OCR values that were either not detectable or just above the detection threshold for all cytokine stimulations (**Figures 5A–E**, right). Similarly to 50,000 cells/well, the seeding density of 75,000 cells/well yielded OCR values that were not consistently detectable for IL-7 alone stimulations (**Figure 5A**, right), and were just above the detection threshold for IL-2 (**Figure 5B**, right), IL-33 (**Figure 5C**, right), IL-7+IL-33 (**Figure 5D**, right), and IL-2+IL-33 treatments (**Figure 5E**, right). The only cell seeding density that gave OCR values consistently within the detection range across all cytokine stimulations was 100,000 cells/well. Furthermore, this seeding density was also the closest to the recommended basal OCR range of 20-160 pmol/min and presented with a post-cytokine stimulation confluency of 80% (**Figures 5A–E**, left). As such, 100,000 cells/well was the cell seeding density chosen for all metabolic assays conducted in this paper (**Figures 5A–E**, left).

### 3.5 Quantification of Mito Stress Assay metrics

As previously addressed in Section 3.4, the Mito Stress Assay is a metabolic assay to evaluate mitochondrial respiration and function. Mitochondrial respiration is driven by the electron transport chain (ETC); a series of five protein complexes (I, II, III, IV, V) located at the interface of the mitochondrial matrix and intermembrane space (39, 42). Through the implementation of compounds such as oligomycin, FCCP, rotenone, and antimycin A, various elements of the ETC can be manipulated to modulate mitochondrial respiration and evaluate individual metrics. The Mito Stress Assay can measure six metrics in total: basal respiration, ATP production, proton (H^+^) leak, maximal respiration, spare respiratory capacity, and nonmitochondrial respiration. Each of the metrics are calculated in reference to the OCR measurements acquired by the Seahorse XF/XFe Analyzer at specific timepoints, and in response to the compound injections that disrupt or facilitate elements of mitochondrial respiration. According to the *Seahorse XF Cell Mito Stress Test Kit User Guide* the (i) basal respiration is measured first and represents the oxygen consumption required to meet the energetic demand of the cell at baseline conditions. (ii) ATP production specifically shows the amount of ATP produced by mitochondrial respiration to meet cellular energy demands. This metric is measured when oligomycin is injected to inhibit ATP synthase (complex V), subsequently reducing the electron flow in the ETC and the subsequent OCR values. (iii) Proton leak can be an indicator of mitochondrial damage but can also be used mechanistically to regulate mitochondrial ATP production. This metric is the difference between the OCR values for basal respiration and ATP-linked respiration. (iv) The maximal respiration demonstrates the maximum rate of respiration that is possible for the cell to achieve and is measured after the injection of FCCP which mimics a physiological “energy demand.” This compound acts as an uncoupling agent that disrupts mitochondrial membrane potential by collapsing the proton gradient, enabling the ETC to operate at maximum capacity to oxidize substrates, such as glucose. (v) The spare respiratory capacity is the cell’s ability to respond to energy demands, which can be used as an indicator for cellular fitness and is calculated by subtracting the basal respiration from the maximum respiration. (vi) Finally, nonmitochondrial respiration is the cellular respiration that continues after the mitochondrial respiration has been inhibited through the injection of rotenone and antimycin A which inhibit complexes I and III, respectively.

**Figure 6:**
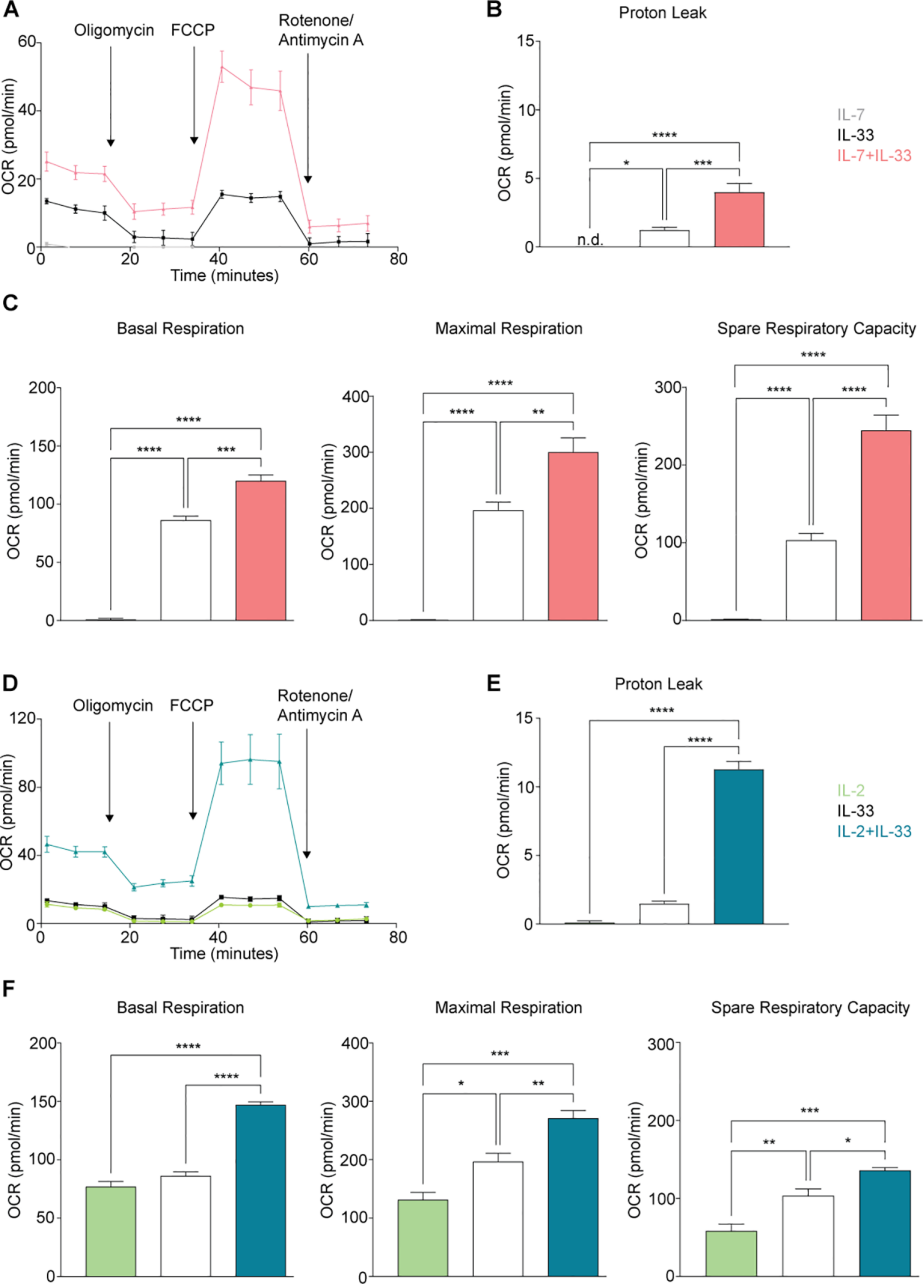
Group 2 innate lymphoid cells (ILC2) elevate oxygen consumption (OCR) rate upon stimulation with activating cytokines. Bone marrow-derived group 2 innate lymphoid cells (ILC2) were seeded into Seahorse XFe96 microplates at a cell density of 100.000 cells/well and stimulated with either IL-7 only, IL-33 only, or a combination of IL-7 and IL-33 **(A-C)**, or with IL-2 only, IL-33 only, or a combination of IL-2 and IL-33 **(D-F)**. All cytokines were used at 10 ng/mL. After 24 hours of cytokine stimulation the Mito Stress Test assay was performed using the Seahorse Analyzer and oxygen consumption rates (OCR) **(A, D)**, proton leak **(B, E)**, basal and maximal respiration as well as the spare respiratory capacities **(C, F)** were determined. Data reporting the treatment with IL-33 are the same for **(A-F)**. Data are shown as average ± standard deviation (SD) and are representative of three independent experiments. Statistical analysis was performed using one-way ANOVA followed by Tukey’s multiple comparisons test (*p ≤ 0.05, **p ≤ 0.01, ***p ≤ 0.001, ****p ≤ 0.0001).

To further understand the intricacies of mitochondrial respiration during steady and activation states, we applied the Seahorse Mito Stress Assay for sort-purified bone marrow-derived ILC2 to evaluate their proton leakage, basal respiration, maximum respiration, and spare respiratory capacity. ILC2 were incubated with IL-7 (**Figure 6A**), IL-2 (**Figure 6D**), IL-33 alone (**Figures 6A, D**), or with combinations of IL-7+IL-33 (**Figure 6A**) or IL-2+IL-33 (**Figure 6D**). After 24 hours of cytokine stimulation, ILC2 were prepped for the Mito Stress Assay (Section 2.8) and evaluated on the Seahorse XF/XFe Analyzer (**Figure 6**). The levels of proton leakage in ILC2 treated with IL-7 alone were lowest of all tested cytokine treatments (**Figures 6B, E**). There was a marked elevation in the levels of proton leakage in the presence of IL-33 alone, as well as with the combined treatment of IL-7+IL-33 (**Figure 6B**). Furthermore, the combined treatment of IL-2+IL-33 demonstrated remarkably higher proton leakage in comparison to IL-2 or IL-33 alone (**Figure 6E**). These observations demonstrate that ILC2 exhibit minimal signs of mitochondrial damage at steady state but were found to exhibit higher levels of proton leakage when synergistically activated by IL-7+IL-33, but especially with IL-2+IL-33.

We established in Section 3.3 that activation of ILC2 results in increased fluorescence of the dye TMRM, a dye that is preferentially acquired by mitochondria in the presence of an active membrane potential. This membrane potential is generated through the ETC during oxidative phosphorylation, and indicates mitochondrial respiration is taking place. With the Mito Stress Assay, we were then able to inhibit or facilitate specific elements of the ETC to evaluate the basal respiration, maximal respiration, and the spare respiratory capacity. These three metrics evaluated in ILC2 treated with IL-7 alone presented with minute OCR levels compared to all other cytokines (**Figure 6C**). There was a marked elevation in OCR levels in the presence of IL-33 alone, as well as with the combined treatment of IL-7+IL-33 (**Figure 6C**) for basal respiration, maximal respiration, and space respiratory capacity. In contrast, ILC2 treated with IL-2 alone already exhibited moderate OCR levels in regards to these three respiratory metrics (**Figure 6F**). The combined treatment of IL-2+IL-33 was significantly higher in basal respiration OCR values in comparison to treatments with IL-2, as well as IL-33 alone (**Figure 6F**). For both the maximal respiration and the spare respiratory capacity, the OCR values in ILC2 treated with IL-2 alone were lower in comparison to all other cytokine treatments (**Figure 6F**). Furthermore, there was marked elevation in the OCR values in the presence of IL-33 alone, as well as with the combined treatment of IL-2+IL-33 for the maximal respiration as well as the spare respiratory capacity. Collectively, these observations demonstrate that ILC2 are utilizing mitochondrial respiration at a minimum during steady state but were found to increase their respiration most when synergistically activated by IL-7+IL-33, as well as IL-2+IL-33.

### 3.6 Quantification of Glycolysis Stress Test metrics

Glycolysis and oxidative phosphorylation are two major and interconnected energy producing processes in the cell. Glycolysis occurs when glucose is metabolized within the cell to generate two molecules of pyruvate, followed by a reducing reaction to form lactate as NADH and then reoxidized to make NAD^+^ (44). This reoxidization occurs in both anaerobic and aerobic glycolysis, however under anaerobic conditions this process occurs in the cytoplasm via lactate dehydrogenase (44). Under aerobic conditions the NADH is first shuttled to the mitochondria and then converted to NAD^+^ before participating in the ETC to generate ATP (44). In either case, the production of lactate in the cytoplasm results in the extrusion of H^+^ into the extracellular medium consequently raising its pH (44). In the Glycolysis Stress Test Assay, the speed at which the extracellular medium becomes acidic due this H^+^ accumulation is measured directly as the Extracellular Acidification Rate (ECAR). According to the *Seahorse XF Glycolysis Stress Test Kit User Guide*, (i) glycolysis is measured first and represents the rate of glycolysis under basal conditions. The ECAR is measured as the injected glucose at saturated levels is converted into pyruvate while producing water, CO_2_, NADH, H^+^, and finally ATP. (ii) The glycolytic capacity is measured after the injection of oligomycin, an ATP synthase inhibitor, effectively shutting down oxidative phosphorylation and shifting energy production entirely to glycolysis. This metric exhibits the highest ECAR measurement and serves as an indication of the cells theoretical maximum rate of glycolysis. (iii) The glycolytic reserve measures the potential or ability of a cell to respond to an energy demand and is defined as the difference between the glycolytic capacity and the glycolytic basal rate. This is measured after the injection of a glucose analogue called 2-DG that inhibits glycolysis by competitively binding the first enzyme in the glycolysis pathway, causing a decrease in the ECAR measurement, which is necessary to prove that the ECAR produced in the experiment was due to glycolysis.

**Figure 7:**
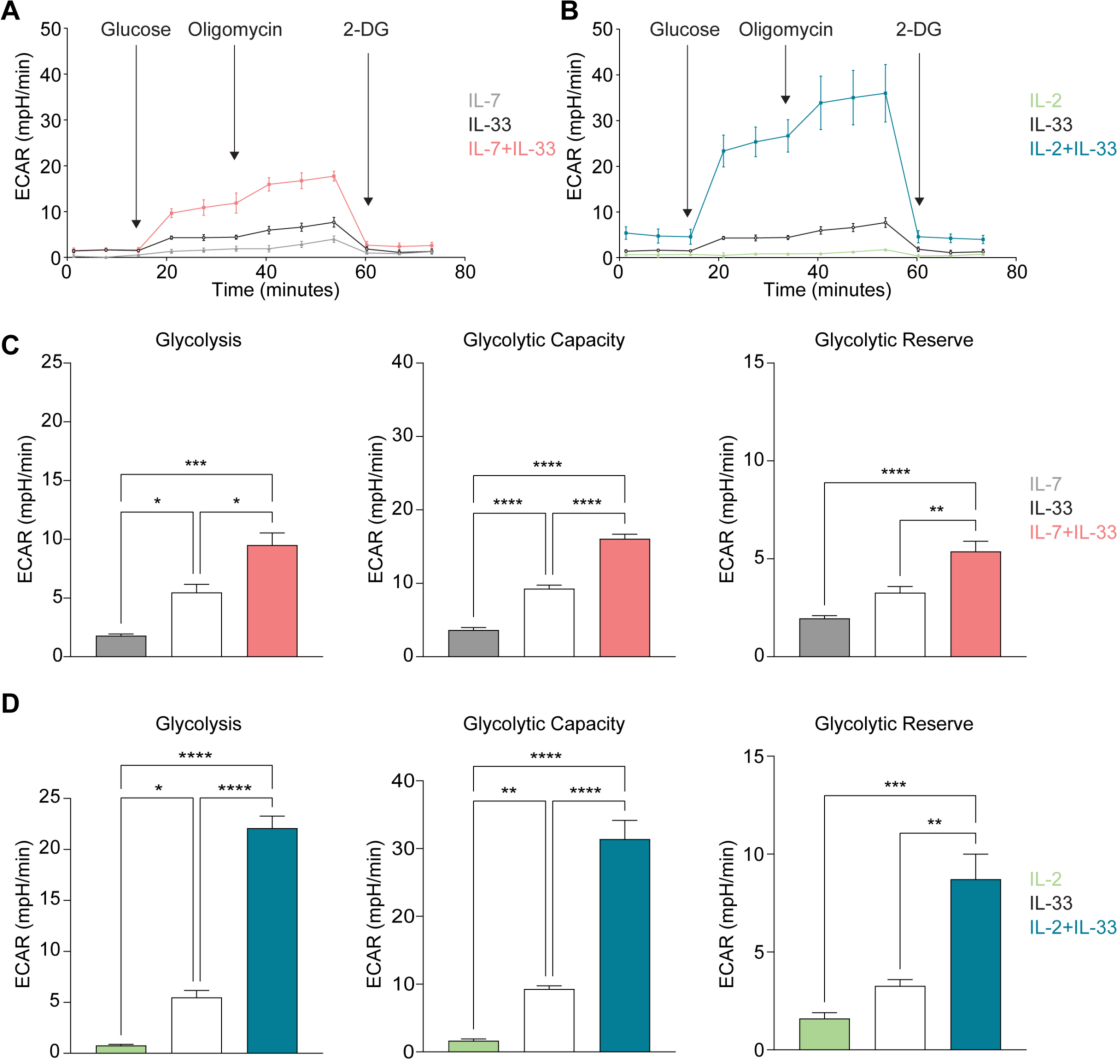
Group 2 innate lymphoid cells (ILC2) increase glycolysis upon stimulation with activating cytokines. Bone marrow-derived group 2 innate lymphoid cells (ILC2) were seeded into Seahorse XFe96 microplates at a cell density of 100.000 cells/well and stimulated with either IL-7 only, IL-33 only, or a combination of IL-7 and IL-33 **(A, C)**, or with IL-2 only, IL-33 only, or a combination of IL-2 and IL-33 **(B, D)**. All cytokines were used at 10 ng/mL. After 24 hours of cytokine stimulation the Glycolysis Stress assay was performed using the Seahorse Analyzer and extracellular acidification rates (ECAR) **(A, B)**, and the levels of glycolysis, glycolytic capacity as well as the glycolytic reserve **(C, D)** were determined (2-Deoxy-D-Glucose (2-DG)). Data reporting the treatment with IL-33 are the same for **(A - D)**. Data are shown as average ± standard deviation (SD) and are representative of three independent experiments. Statistical analysis was performed using one-way ANOVA followed by Tukey’s multiple comparisons test (*p ≤ 0.05, **p ≤ 0.01, ***p ≤ 0.001, ****p ≤ 0.0001).

To investigate how cytokines known to drive ILC2 effector functions influence glycolysis during steady and activation states, we optimized the Seahorse Glycolysis Stress Test for sort-purified bone marrow-derived ILC2 to analyze basal glycolysis, glycolytic capacity, and glycolytic reserve. To this end, ILC2 were incubated with IL-7, IL-2, IL-33 alone, or with combinations of IL-7+IL-33 or IL-2+IL-33. After 24 hours of cytokine stimulation, ILC2 were prepped for the Glycolysis Stress Test (Section 2.8) and evaluated on the Seahorse XF/XFe Analyzer (**Figures 7A, B**). The basal glycolytic rate (Glycolysis; left) and the glycolytic capacity (center) in ILC2 treated with IL-7 (**Figure 7C**) or IL-2 alone (**Figure 7D**) were significantly lower in comparison to all other cytokine treatments. There was a marked elevation in both metrics in the presence of IL-33 alone, as well as with the combined treatments of IL-7+IL-33 (**Figure 7C**) and IL-2+IL-33 (**Figure 7D**). In contrast, while there was no difference between IL-7 (**Figure 7C**) or IL-2 alone (**Figure 7D**) compared to IL-33 alone when measuring the glycolytic reserve values (right), the combined cytokine treatments of IL-7+IL-33 (**Figure 7C**) and IL-2+IL-33 (**Figure 7D**) were significantly higher in comparison to all other cytokine treatments. These observations demonstrate that ILC2 are utilizing glycolysis at a minimum and exhibit a low glycolytic capacity during steady state (IL-7 or IL-2 only), but were found to increase the glycolytic rate and capacity when stimulated with IL-33, and the most when synergistically activated by IL-7+IL-33, or by IL-2+IL-33.

### 3.7 SCENITH analysis

We next took advantage of a recently described method referred to as Single Cell ENergetic metabolism by profilIng Translation inHibition (SCENITH), which enables us to determine metabolic dependencies and capacities in their entirety at the single-cell level. While SCENITH has been shown to be as reliable and comparable to other well-established techniques such as Seahorse, it is an especially well-suited approach to analyze rare cells like ILC2s, as SCENITH requires far fewer cells and is less dependent on cell density (45). SCENITH incorporates the detection of puromycin as its primary readout, providing a proxy measurement for global translation and protein synthesis (46). (45) The contribution of the glycolytic or OXPHOS metabolic pathways in terms of energy production can be assessed by analyzing protein synthesis in the presence of inhibitors, where glycolysis is inhibited by 2-deoxyglucose (2-DG), OXPHOS with oligomycin, or both to inhibit all energy production. Protein synthesis is an energy demanding process, therefore SCENITH equates decreased protein synthesis with the interruption of ATP production caused by the addition of these inhibitors. In all, SCENITH quantifies four metrics: glucose dependence, mitochondrial dependence, glycolytic capacity, and fatty acid oxidation (FAO) and amino acid oxidation (AAO) capacity (Section 2.12). While the total level of translation correlates with the global metabolic activity of the cells, the dependency parameters underline essential cellular pathways. In contrast, “capacity” is the inverse of dependency, which reveals the maximum compensatory capacity to exploit alternative pathway/s when one is inhibited.

**Figure 8:**
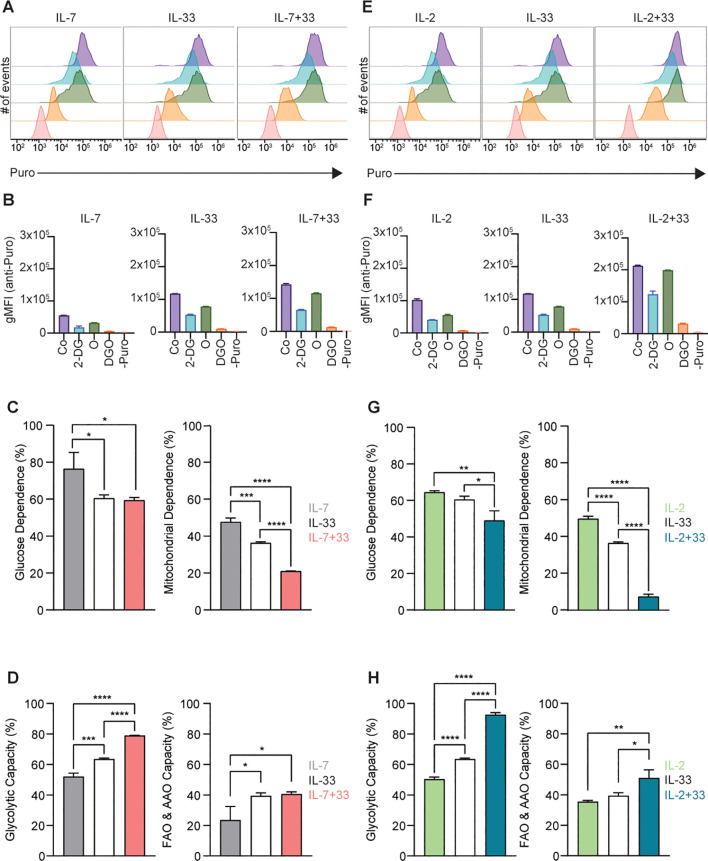
SCENITH analysis of group 2 innate lymphoid cells (ILC2). **(A-F)** SCENITH (Single Cell ENergetIc metabolism by profilIng Translation inHibition) analysis was performed by stimulating bone marrow-derived group 2 innate lymphoid cells (ILC2) with either IL-7 only, IL-33 only, or a combination of IL-7 and IL-33 **(A-D),** or IL-2 only, IL-33 only, or a combination of IL-2 and IL-33 **(E-H)**. All cytokines were applied at 10 ng/mL. After 24 hours of cytokine stimulation cells were either left untreated as control (Co) or incubated with 2-Deoxy-D-Glucose (2-DG), Oligomcyin (O), or a combination of 2-DG and O (DGO) for 30 minutes. Subsequently, cells were treated for 15 minutes with puromycin followed by intracellular staining with an anti-puromycin antibody. Cells that were not treated with puromycin were used as a negative control (-Puro). ILC2 were then analyzed by flow cytometry **(A, E),** geometric mean intensities (gMFI) of the anti-puromycin staining acquired **(B, F),** and gMFI values used to determine glucose dependence and mitochondrial dependence **(C, G)**, as well as glycolytic and fatty acid oxidation (FAO) and amino acid oxidation (AAO) capacities **(D, H)**. Data reporting the treatment with IL-33 are the same for **(A-H)**. Data are shown as average ± standard deviation (SD) and are representative of three independent experiments. Statistical analysis was performed using one-way ANOVA followed by Tukey’s multiple comparisons test (*p ≤ 0.05, **p ≤ 0.01, ***p ≤ 0.001, ****p ≤ 0.0001).

The global level of translation was low in ILC2s stimulated with IL-7 (**Figures 8A, B**) or IL-2 (**Figures 8E, F**) alone, gradually increased when treated with IL-33 alone, but protein synthesis was highest when ILC2s were stimulated with IL-7+IL-33 (**Figures 8A, B**) and IL-2+IL-33 (**Figures 8E, F**). These data also revealed that ILC2s stimulated with IL-7+IL-33 (**Figure 8C**) and IL-2+IL-33 (**Figure 8G**) exhibited a significantly lower dependence on glucose and mitochondria compared to ILC2s treated with IL-7 (**Figure 8C**) or IL-2 (**Figure 8G**) alone. ILC2s treated with IL-33 alone presented a reduced dependence on glucose and mitochondria compared to IL-7 (**Figure 8C**), whereas only a reduced dependence on mitochondria was observed between IL-33 treated cells and IL-2 (**Figure 8G**) alone. Similarly, ILC2s treated with IL-2+IL-33 (**Figure 8G**) exhibited an even lower dependence on glucose and mitochondria compared to IL-2 (**Figure 8G**), whereas only a reduced dependence on mitochondria was observed between IL-7+IL-33 treated cells and IL-33 (**Figure 8G**) alone. Conversely, compared to the glycolytic capacity of ILC2 treated with IL-7 (**Figure 8D**) or IL-2 (**Figure 8H**), a marked elevation was observed when cells were stimulated with IL-33, and again when stimulated with IL-7+IL-33 (**Figure 8D**) or IL-2+IL-33 (**Figure 8H**). When evaluating the FAO & AAO capacity, ILC2s treated with IL-7 alone (**Figure 8D**) exhibited the lowest capacity compared to all other cytokine conditions, whereas those stimulated with IL-2+IL-33 (**Figure 8H**) exhibited a higher capacity compared to all other conditions. Collectively, these data demonstrate that ILC2s exhibit great metabolic flexibility in the presence of IL-33, but even more so when stimulated with the synergistic combination of activating cytokines IL-7+IL-33, as well as IL-2+IL-33.

### 3.8 Characterization of ATP production via Agilent™ Seahorse Real-Time ATP Rate Assay

**Figure 9:**
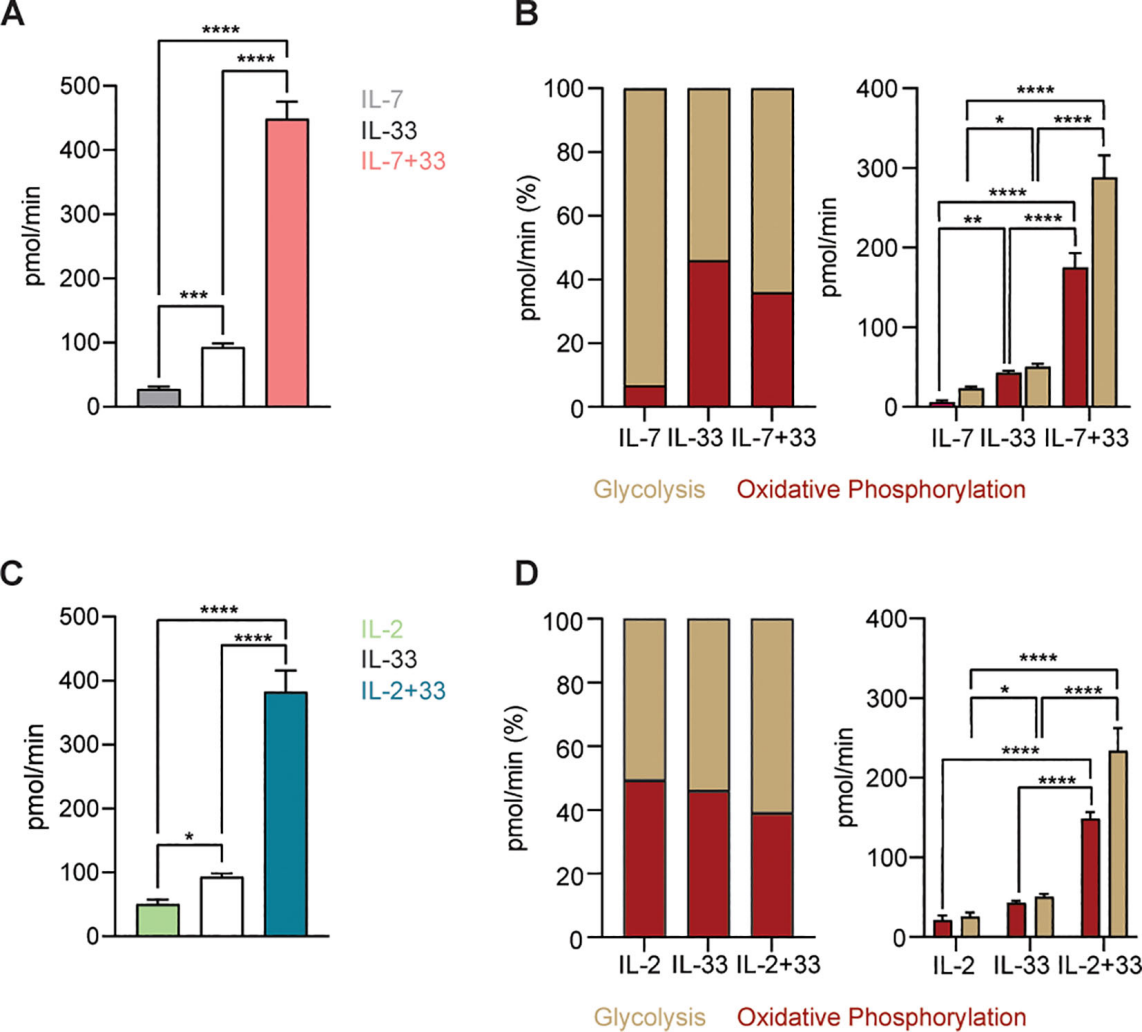
Group 2 innate lymphoid cells (ILC2) augment ATP production upon stimulation with activating cytokines. **(A-D)** To determine ATP production bone marrow-derived group 2 innate lymphoid cells (ILC2) were seeded into Seahorse XFe96 microplates at a cell density of 100.000 cells/well and stimulated with either IL-7 only, IL-33 only, or a combination of IL-7 and IL-33 **(A, B)**, or with IL-2 only, IL-33 only, or a combination of IL-2 and IL-33 **(C, D)**. All cytokines were used at 10 ng/mL. After 24 hours of cytokine stimulation the Real-Time ATP Rate assay was performed using the Seahorse Analyzer. ATP production from oxidative phosphorylation as well as glycolysis were analyzed and depicted as a proportion of 100% and as absolute values. Data reporting the treatment with IL-33 are the same for **(A-D)**. Data are shown as average ± standard deviation (SD) and are representative of three independent experiments. Statistical analysis was performed using one-way ANOVA followed by Tukey’s multiple comparisons test (*p ≤ 0.05, **p ≤ 0.01, ***p ≤ 0.001, ****p ≤ 0.0001).

In our final analysis step, we aimed to quantify absolute ATP production levels of ILC2 using the Agilent™ Seahorse XF ATP Real-Time rate assay, which also measures and quantifies the rate of ATP production from the glycolytic and mitochondrial system simultaneously (Section 2.10). As expected, stimulations with IL-7 (**Figure 9A**) or IL-2 alone (**Figure 9C**) yielded low ATP production rates. While treatment of ILC2 with IL-33 slightly increased ATP production, a massive elevation of energy production was observed when cells were activated with IL-7+IL-33 (**Figure 9A**) or IL-2+IL-33 (**Figure 9C**). ATP generation by ILC2 stimulated by only IL-7 was largely driven by glycolysis (**Figure 9B**). In contrast, IL-2 as well as IL-33-driven energy production comparably relied on OXPHOS as well as glycolysis (**Figures 9B, D**). Compared to the IL-33 stimulation only, glycolysis-mediated ATP production frequencies increased only slightly when ILC2 were treated with combinations of IL-7+IL-33 (**Figure 9B**) or IL-2+IL-33 (**Figure 9D**). However, the massive increase in ATP upon IL-7+IL-33 (**Figure 9B**) or IL-2+IL-33 (**Figure 9D**) stimulation, compared to IL-7, IL-2, or IL-33 only treatments, was driven by glycolysis as well as OXPHOS.

## 4 Discussion

ILC2 exert critical functions to ensure tissue barrier integrity, driving and reinforcing immunological protection by orchestrating innate as well as adaptive immune processes. However, when deregulated, ILC2 have been shown to contribute to the pathogenesis of several chronic inflammatory barrier disorders through the release of large quantities of type 2 signature cytokines including IL-4, IL-5 and IL-13. Although recent studies started to uncover critical components of the metabolic wiring of ILC2, many aspects remain elusive. This is largely due to the rarity of ILC2 in tissues and their limited expansion in culture posing a challenge on obtaining sufficient cell counts to carry out large-scale experiments. Moreover, there is a lack of accessibility of rapid assays to study the intricacies of metabolic pathways. Applying our recently described protocol to expand murine bone marrow-derived ILC2 (23, 33), we detail here a framework of experimental approaches to study key immunometabolic states utilizing flow cytometry, SCENITH, as well as the Seahorse platform.

We provide an in-depth protocol of how to use ILC2 for studies applying the Seahorse platform, where drug treatments can be delivered to the cells at specific targets and timepoints to further elucidate the metabolic identities of ILC2. While the information from Seahorse assays is valuable, they require special equipment as well as proprietary kits and consumables, thereby limiting the number of researchers accessing this platform due to financial and accessibility restrictions. We therefore further detail flow cytometry-based assays, including SCENITH, that can be rapidly implemented for metabolic studies of ILC2. Seahorse and SCENITH assays exhibit both a high degree of accuracy and provided complementary insights into the metabolic programming of ILC2. Comparatively, the high values for glycolytic and OXPHOS capacities in ILC2 treated with IL-7+IL-33 and IL-2+IL-33 found in Seahorse were corroborated by those found using SCENITH. In fact, the trend in values between single cytokine stimulations and synergistic cytokine treatments were consistent between the two platforms. In both Seahorse and SCENITH approaches, the IL-2 alone conditions exhibited values that were higher than the stimulations with IL-7 alone. Although overall SCENITH is more user-friendly and accessible to the research community, the Seahorse platform provides additional insights that are highly specific. As such, we would recommend that, if possible, researchers should use SCENITH to corroborate and bolster insights revealed by Seahorse.

Our applied framework of experimental approaches combining flow cytometry assays with proprietary metabolic platforms demonstrates that the utilization of glucose is markedly elevated upon cytokine-mediated activation of ILC2. In parallel, we reveal increases in mitochondrial biogenesis (PGC-1α), mitochondrial mass as well as mitochondrial membrane potential upon ILC2 activation. Our study reveals that ILC2 take up moderate levels of glucose, exhibit low levels of mitochondrial respiration, and utilize glycolysis at a minimum rate during steady state (IL-7 or IL-2 alone), but gradually increase their glucose metabolism, as well as OXPHOS, upon activation with IL-33. Interestingly, while glycolysis is not heavily utilized at steady state, the proportion of ATP produced by ILC2 is almost exclusively from glycolysis in the presence of IL-7. In contrast, IL-2 treated ILC2 consistently present with a nearly 50:50 ratio of glycolysis- and OXPHOS-mediated ATP production. Overall, mitochondrial respiration if highly elevated, with OXPHOS producing more ATP than glycolysis. In fact, IL-2+IL-33 exhibited the highest values for mitochondrial respiration (TMRM) and basal respiration (Seahorse). Increased mitochondrial respiration is typically correlated with an increased demand for energy which can induce the production of ROS that damage mitochondrial integrity, consequently stimulating mitochondrial biogenesis. Concurrently, we observe that ILC2 treated with IL-2+IL-33 present the highest degree of proton leak and mitochondrial respiration. The mitochondrial mass in ILC2 treated with IL-33 was higher than that of those treated with IL-7 or IL-2 alone. However, this mass decreased in the synergistic combination of these cytokines. It is possible that ILC2 treated with IL-33 increased their mitochondria production to meet the energy demand, but the increased respiration caused damage to the mitochondria and the overall mass decreased as a consequence. While PGC-1α levels as well as mitochondrial mass increased with IL-33 stimulation compared to homeostatic cytokine treatments with IL-7 or IL-2 alone, synergistic cytokine activation did not yield an elevation of values above those obtained with IL-33 only. In contrast, stimulations with IL-7+IL-33 or IL-2+IL-33 markedly elevated their mitochondrial potential, indicative of mitochondrial respiration. These findings were further complemented by Seahorse Real-Time ATP Rate analysis as well as SCENITH, revealing that synergistic activation of ILC2 with IL-7+IL-33 or IL-2+IL-33 led to markedly higher ATP production and protein synthesis and elevated glycolytic and FAO & AAO capacities. Collectively, this demonstrates that the increase in mitochondrial potential upon synergistic activation with IL-7+IL-33 or IL-2+IL-33 translates into elevated energy production, leading to synergistic cytokine production and proliferation of ILC2 (23, 33). Interestingly, SCENITH analysis further demonstrated that ILC2 treated with IL-7+IL-33 or IL-2+IL-33 have reduced glucose and mitochondrial dependence, suggesting a high metabolic flexibility of ILC2 when activated by IL-33, but especially when primed by IL-7+IL-33, or IL-2+IL-33. Taken together, these findings indicate that metabolic substrate accessibility and availability is a main driver in the metabolic wiring of ILC2, which will need further experimental interrogation in future studies.

## Data Availability

The raw data supporting the conclusions of this article will be made available by the authors, without undue reservation.

## Supplementary material

Supplementary Figure 1 Gating strategies for the isolation of murine bone marrow-derived group 2 innate lymphoid cell (ILC2). Debris and doublets were excluded and murine bone marrow-derived ILC2 precursors were defined and isolated by flow cytometric sorting as lineage-negative, c-kit^-^Sca-1^+^CD25^+^ cells.
