# Supplementary figures and images for: Immunometabolic analysis of primary murine group 2 innate lymphoid cells: a robust step-by-step approach
Source: Front Immunol. 2025 Mar 13;16:1545790. doi: 10.3389/fimmu.2025.1545790 (PMC11966487; doi:10.3389/fimmu.2025.1545790)

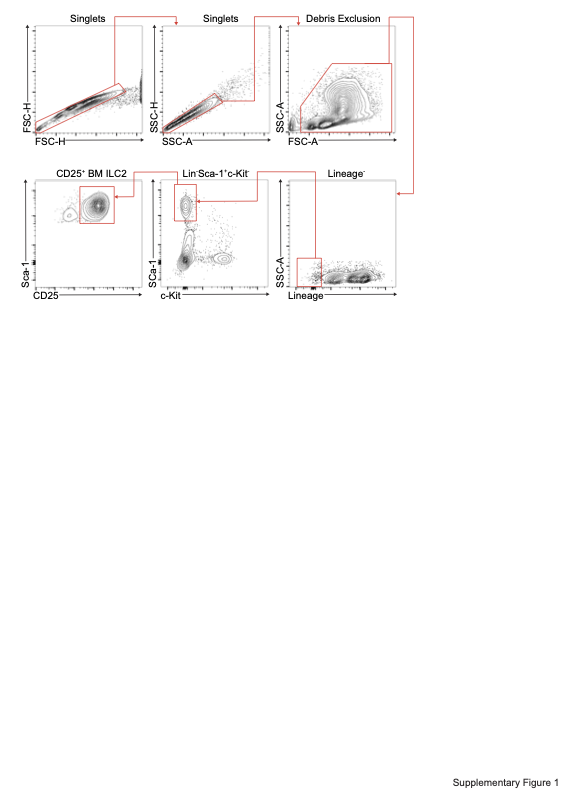

Supplement: Supplementary Figure 1 — Gating strategies for the isolation of murine bone marrow-derived group 2 innate lymphoid cell (ILC2). Debris and doublets were excluded and murine bone marrow-derived ILC2 precursors were defined and isolated by flow cytometric sorting as lineage-negative, c-kit-Sca-1+CD25+ cells. [file Image1.tiff]
